# Elevated Connectivity During Language Processing Is Associated With Cognitive Performance in SeLECTS

**DOI:** 10.1002/acn3.70369

**Published:** 2026-04-21

**Authors:** Wendy Qi, Katharine Lee, Kerry C. Nix, Miguel Menchaca, Xiwei Shé, Lorelei Santa Maria, Wei Wu, Zihuai He, Fiona M. Baumer

**Affiliations:** ^1^ Department of Neurology and Neurological Sciences Stanford University Palo Alto California USA; ^2^ Department of Psychiatry & Behavioral Sciences Stanford University Palo Alto California USA

**Keywords:** Benign Epilepsy with Centrotemporal Spikes (BECTS), Childhood Epilepsy with Centrotemporal Spikes (CECTS), electroencephalogram, functional connectivity, language processing, pediatric epilepsy, phonological awareness, Rolandic Epilepsy

## Abstract

**Objective:**

Self‐Limited Epilepsy with Centrotemporal Spikes (SeLECTS) is associated with language impairments despite seizures originating in the motor cortex, suggesting aberrant cross‐network interactions. Here we tested whether functional connectivity in SeLECTS during language tasks predicts language performance.

**Methods:**

We recorded high‐density EEG from right‐handed children with SeLECTS (*n* = 31) and age‐matched controls (*n* = 32) during verb generation, repetition, and resting tasks. Phonological awareness was assessed using the Comprehensive Test of Phonological Processing‐2. Connectivity between bilateral motor cortices and language regions (the left inferior frontal and superior temporal cortices and their right hemisphere homologs) was measured using the weighted Phase Lag Index (wPLI).

**Results:**

Children with SeLECTS demonstrated significantly elevated connectivity between motor and language regions during language processing. Motor‐to‐frontal connectivity was higher in SeLECTS during both verb generation and repetition tasks. Frontal‐to‐temporal connectivity was elevated specifically during verb generation. Exploratory analyses suggest high interhemispheric connectivity (between the left and right hemispheres) during language tasks is associated with poor phonological awareness in children with SeLECTS, but not controls. Together, we found that children with SeLECTS exhibited pathologically elevated connectivity between motor and language networks that was strongly associated with impaired phonological awareness.

**Interpretation:**

These findings establish task‐specific connectivity abnormalities as a characteristic feature of SeLECTS and suggest interhemispheric connectivity patterns may relate to language outcomes, warranting further investigation as potential targets for therapeutic neuromodulation.

## 1 Introduction

Self‐limited Epilepsy with Centrotemporal Spikes (SeLECTS) is a common pediatric epilepsy syndrome characterized by frequent spike waves in the motor cortex that are potentiated in sleep [1]. Children with SeLECTS have mild to moderate language impairments [2, 3, 4], often lagging a full standard deviation behind typically developing peers. In addition to causing language dysfunction, SeLECTS affects development of the language network—spatially distant brain regions whose coordinated activity subserves language function [5]. Whereas most typically developing children show predominantly left‐hemisphere language processing by school age [6], children and adolescents with a history of SeLECTS show bilateral temporal and frontal activity during language tasks [7, 8].

Frequent centrotemporal spikes in SeLECTS contribute to language dysfunction, but the mechanism by which they do this is unclear. Spike frequency does not consistently predict language performance [4]. Instead, spikes' impact on aberrant functional connectivity remains a likely but unproven hypothesis for language dysfunction. Studies using multiple modalities [9, 10, 11] find that spikes acutely alter functional connectivity between epileptic motor cortices and the language network [12, 13, 14], including the left inferior frontal cortex (IF, Broca's area), and the left superior temporal cortex (ST, Wernicke's area). The greater the acute change induced by each spike, the worse the language function [15], suggesting that susceptibility to spike‐induced connectivity disruption is a marker of language vulnerability. Children with SeLECTS also have chronically elevated connectivity between spikes, particularly in sleep [14]. Critically, EEG‐based connectivity has only been examined in resting‐state recordings in SeLECTS, even though studies from other patient populations suggest that studying connectivity during language processing can reveal unique abnormalities not detectable at rest [16]; functional connectivity during language tasks in children with SeLECTS has not yet been explored with EEG. Elucidating the role of connectivity in language is clinically relevant, as recent advances in noninvasive neuromodulation techniques, including transcranial magnetic stimulation, have demonstrated the feasibility of selectively modulating cortical connectivity in pediatric populations, opening unprecedented opportunities for targeted cognitive therapeutic interventions in epilepsy [17].

To test the role of connectivity in language, we compared connectivity in children with and without SeLECTS as they performed three tasks (verb generation, word repetition, and resting state) [18] and assessed if connectivity was associated with standardized language scores, focusing on phonological awareness as it is consistently reported as abnormal in SeLECTS [4]. We measured connectivity during spike‐free trials between six regions: the bilateral motor cortices, left IF and ST language cortices, and right IF and ST homologs using the weighted Phase Lag Index (wPLI), a phase‐based connectivity measure robust against volume conduction [19]. We focused on the theta (4 to < 8 Hz) frequency band because theta has been most strongly implicated in language, learning and memory [20, 21]. We also explored connectivity in other frequency bands as wide‐ranging abnormalities have been reported in SeLECTS during the resting state [22]. We hypothesized that spikes drive excessive hyperconnectivity between epileptic motor and language regions and that, in turn, this pathologic hyperconnectivity interferes with typical language network development, leading to reduced IF to ST connectivity. Furthermore, we expected that high motor‐to‐language connectivity and low IF‐to‐ST connectivity would be associated with worse phonological awareness.

## 2 Methods

### 2.1 Subjects

Children with a diagnosis of SeLECTS [1] per ILAE criteria (history of focal seizures during sleep and centrotemporal spikes) were recruited from Lucile Packard Children's Hospital or surrounding child neurology practices. Typically‐developing children with no history of seizures (except simple febrile seizures) or anti‐seizure medication (ASM) use were recruited from the community. Inclusion criteria for both groups included being right‐handed, 5–13 years old, and born after 35 weeks' gestation. Children all spoke English though could be bilingual. Exclusion criteria consisted of a history of severe neurologic or systemic problems (i.e., stroke, traumatic brain injury, cardiac or oncologic disease). Children with attention‐deficit/hyperactivity disorder (ADHD) were not excluded from either group [23]. Data were collected between February 2021 and November 2023. This study was approved by Stanford University's Institutional Review Board, with informed consent from parents and assent from children.

### 2.2 Clinical Information

We recorded age, sex, number of lifetime seizures, presence of seizures in sleep only vs. sleep and wakefulness, ASM usage (type), Edinburgh handedness inventory score [24], bilingual status, socioeconomic status (Hollingshead index score) [25], and ADHD history.

### 2.3 Experimental Setup

Participants sat in front of a computer monitor and speaker in a sound‐proof room (Figure 1). High‐density EEG was recorded with the Electrical Geodesics NetStation system using 128‐channel saline‐based caps (sampling rate 1000 Hz) with impedances maintained below 50 kΩ. Stimuli were presented using E‐Prime software. Participants completed three tasks (verb generation, repetition, and resting) while staring at a fixation cross to limit eye movement.

**FIGURE 1:**
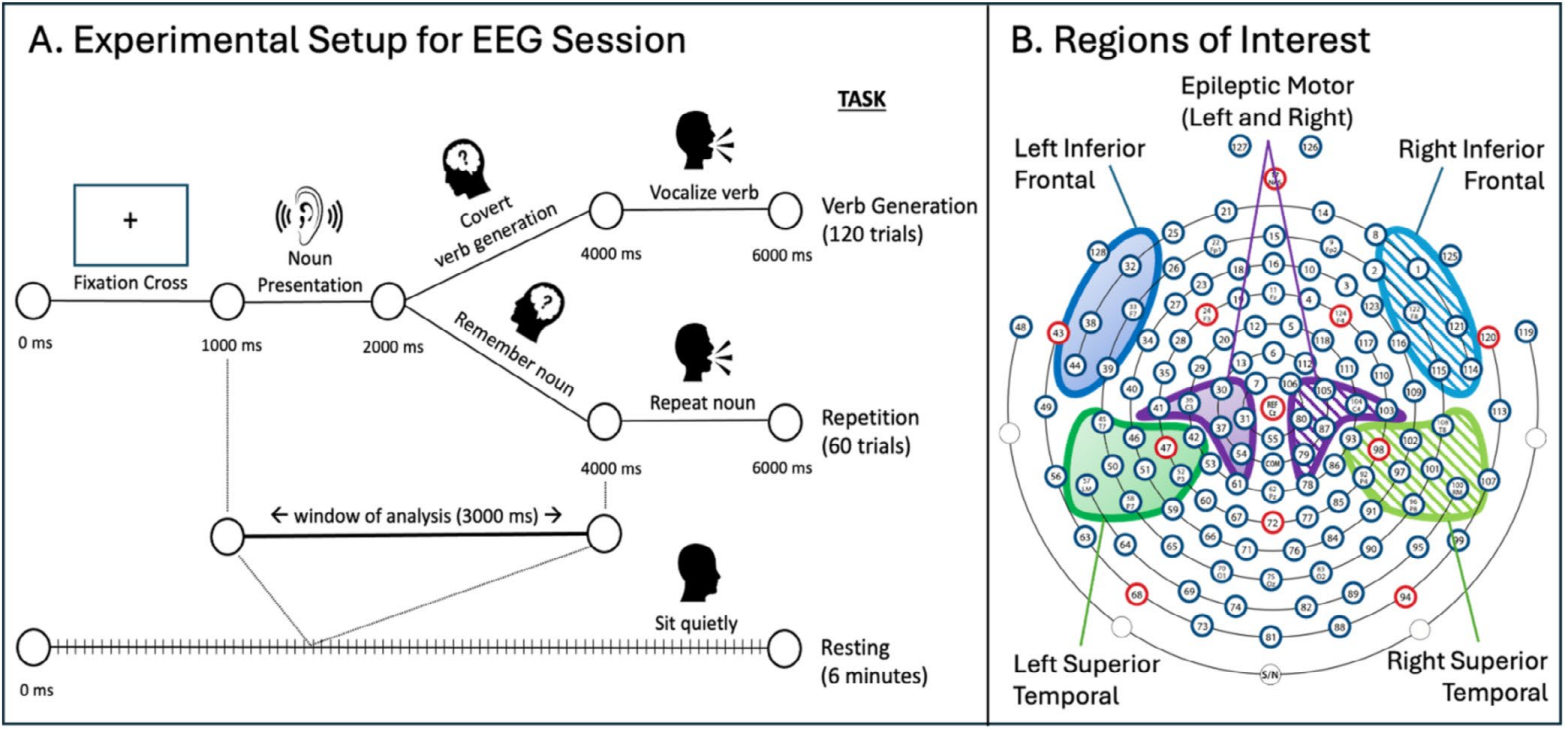
Experimental setup. (A) Participants completed three tasks (verb generation, repetition, and resting) while high‐density EEG was recorded. (B) Topographic plot showing electrodes in each region of interest: Inferior frontal (blue), superior temporal (green), and motor (purple) regions with shading indicating hemisphere (solid = left; dashed = right).

The verb generation and repetition tasks began with an auditory presentation of a noun (500–1000 ms in duration) chosen from the Children's Printed Word Database [26]. Nouns in each block were matched in terms of duration, syllables, and word frequency. The verb generation task, a validated method for activating the language network [27, 28], consisted of hearing a noun and silently generating an associated “verb or action word.” The repetition task consisted of remembering a noun. After 3 s, participants were cued to vocalize the verb or repeat the noun; this overt response period was used only to confirm task participation and was not included in connectivity analyses. The repetition task was chosen as a control as it requires working memory and motor planning but lacks the semantic retrieval of verb generation [18]; furthermore, verb generation (but not word repetition) evokes lateralized EEG changes [18, 29]. Two 60‐trial blocks of verb generation and one 60‐trial block of repetition were conducted. During the resting task, participants sat quietly with their eyes open while looking at the fixation cross for two 3‐min blocks. Block order was randomized on a per participant basis.

### 2.4 Task Performance

Verb generation responses were coded as correct (appropriate verb); retrieval error (no or non‐verb response); or task error (repeated noun). Repetition responses were coded as correct or incorrect.

### 2.5 Neuropsychological Testing

Testing was performed on a separate day from the EEG by research coordinators (KN, WQ) who had been trained by clinicians. Testing occurred over Zoom given COVID‐19 restrictions and was appropriately scored as per validated online testing methods [30]. The assessment included the Comprehensive Test of Phonological Processing (CTOPP‐2) [31] Phonological Awareness composite and the Wechsler Abbreviated Scale Intelligence (WASI‐II) [32] two‐subtests form. Phonological awareness was the primary neuropsychological outcome of interest as it is most consistently altered in children with SeLECTS [4, 33].

### 2.6 Epilepsy Severity

There is not a standardized method for grading epilepsy severity in SeLECTS, but we chose measures based on the GASE survey [34]. We measured the number of lifetime seizures, presence of seizures during sleep only (vs. sleep and wakefulness), presence of seizures lasting for more than 5 min, and presence of seizures with secondary generalization, as these factors are important for patient quality of life and could be reliably extracted from medical records. We also categorized patients based on whether spikes were present or absent during the research EEG, as this indicates spike activity during wakefulness.

## 3 Data Analysis

### 3.1 Preprocessing

Data were preprocessed in MATLAB (version 2021b) using the Maryland Analysis of Developmental EEG (MADE) pipeline, designed specifically for pediatric EEG preprocessing [35]. For each participant, EEG from all tasks was concatenated, downsampled to 500 Hz, and bandpass filtered (> 1 and < 50 Hz). No frequency‐specific pre‐filtering was applied during preprocessing. Instead, wPLI was calculated for specific frequency bands (theta: 4–8 Hz, alpha: 8–12 Hz, beta: 13–30 Hz, gamma: 30–80 Hz) using spectral decomposition during the connectivity analysis phase. Channel rejection criteria (*Z*‐score > 3 for Hurst exponent, inter‐channel correlation, and variance) follow the validated MADE preprocessing pipeline designed specifically for developmental EEG data [35]. Independent component analysis (ICA) decomposition was used to identify and remove artifactual independent components (ICs) [35]. For the verb generation and repetition tasks, three‐second epochs were extracted starting at noun onset, capturing the period of silent/covert cognitive processing and excluding the period of overt speech. Resting data was divided into three‐second, nonoverlapping epochs. Epochs in which the voltage of both ocular electrodes and > 10% of non‐ocular electrodes exceeded ±150 μV were rejected. In retained epochs, individual channels exceeding the voltage threshold were interpolated on an epoch‐by‐epoch basis. Finally, channels rejected earlier in preprocessing were also interpolated. EEG signals were re‐referenced to the average of all channels during preprocessing [35]. A board‐certified epileptologist (FMB) manually reviewed remaining epochs and excluded those containing spikes, as spikes acutely increase connectivity [10, 14] and our focus was connectivity elicited by language processing.

Since connectivity estimates are sensitive to epoch number [36], we aimed to keep trial numbers consistent across subjects and tasks while maximizing data inclusion. Tasks with fewer than 20 epochs after preprocessing [36] were excluded. Afterwards, all epochs up to a maximum of 50 epochs per task were included in connectivity calculations with a random number generator used to reduce to 50 if needed.

### 3.2 Extraction of Connectivity Values

Connectivity was calculated using an undirected phase‐based measure of brain connectivity, called the wPLI [19], a robust measure of phase synchronization and connectivity minimally affected by volume conduction [19, 36, 37] that is elevated in SeLECTS [14]. We calculated wPLI with cross‐spectral density in Fieldtrip's connectivity toolbox implementation in MATLAB, as previously published [38]. wPLI was calculated using 3000 ms nonoverlapping epochs with identical spectral parameters across all frequency bands. Cross‐spectral density was computed using a multitaper method with frequency‐dependent taper parameters (four tapers for theta band, bandwidth 2 Hz; adjusted proportionally for other bands). To address non‐stationarity concerns inherent in scalp EEG, we implemented multiple preprocessing steps: spike‐epoch exclusion by epileptologist review, voltage‐based artifact rejection (±150 μV threshold), and selection of the most artifact‐free epochs up to a maximum of 50 per condition. A wPLI value was calculated first for each epoch and then averaged across all epochs to obtain a single connectivity value per electrode pair (1953 unique electrode pairs).

### 3.3 Regions of Interest

Regions of interest included the bilateral motor cortices where spikes originate, the left IF and ST regions, and homologous right‐sided IF and ST regions [39]. The ST regions also included electrodes overlaying the angular and supramarginal gyri which are important for receptive language [40]. Electrodes corresponding to each region were chosen using standard electrode positions [39]. wPLI values from electrode pairs spanning these regions of interest (e.g., wPLI between each left motor and each left IF electrode pair) were averaged to form a single region‐to‐region connectivity value, a technique commonly used to enhance signal‐to‐noise ratio [41]. Prior to this averaging step, we examined distributions of the electrode‐pair wPLI values that made‐up the region‐to‐region connectivity value and confirmed a low coefficient of variation (< 0.3) supported this averaging approach [42]. We computed wPLI for 12 region‐to‐region pairs representing unique connections between bilateral motor, ST and IF regions.

### 3.4 Statistical Analysis

Analyses were performed with Statistical Analysis System (SAS) OnDemand for Academics [43]. Main analyses compared children with SeLECTS to typically developing controls. Exploratory analyses assessed the impact of ASM on connectivity by dividing SeLECTS participants into two subgroups depending on presence (SeLECTS+ASM) or absence (SeLECTS‐ASM) of daily ASM use. Independent *t*‐tests or one‐way ANOVA for continuous variables and chi‐squared for categorical variables were used to compare groups on: age, sex, handedness, Hollingshead SES Index, IQ, and CTOPP‐2 Composite Score. Groups were also compared on task performance and EEG data quality metrics (rejected channels, epochs, ICs).

#### 3.4.1 Group Differences in Connectivity

We first compared connectivity in children with and without SeLECTS, hypothesizing that children with SeLECTS would have higher connectivity between epileptic motor, IF and ST cortices during all tasks and lower connectivity between the left IF and ST cortices specifically during the verb generation task. Our primary analyses focused on connectivity in the theta frequency band (4 to < 8 Hz), which was empirically chosen since it is the frequency band most strongly associated with expressive language processing, word reading, memory, and hippocampal activity [20, 21]. Since subjects had three connectivity measurements (one per task), we used a generalized estimating equation (GEE) with an independent correlation matrix [44] to account for repeated measures and correlation within individuals. We fit a model with theta connectivity as the dependent variable and group (SeLECTS/controls), task (verb generation/repetition/resting), and the group by task interaction as independent variables, with adjustment for age and sex. We performed analyses stratified by task. We ran separate models for each of the 12 region‐to‐region connectivity pairs and considered *p* < 0.0042 significant as per Bonferroni correction. To explore the ASM effect, we fit GEE models in which the group variable was three levels (Controls/SeLECTS+ASM/SeLECTS‐ASM). We also assessed if there was a difference in epilepsy severity in the SeLECTS+ASM vs. SeLECTS‐ASM groups using *t*‐test and chi‐square tests for continuous and categorical variables respectively.

#### 3.4.2 Association Between Connectivity and Phonological Awareness

To test if connectivity is associated with phonological awareness, we fit a GEE model with CTOPP‐2 Composite score as the dependent variable and connectivity, group (SeLECTS/controls), task (verb generation/repetition/resting), and group by task interaction as independent variables, adjusting for age and sex. We conducted analyses stratified by group and task. We hypothesized that children with SeLECTS have different connectivity patterns than controls and modeled within groups so as not to obscure relevant group‐specific relationships. We ran separate models for each region‐to‐region pair, again using *p* < 0.0042 as the significance threshold.

### 3.5 Supplementary Analyses

First, we explored group differences in connectivity in other frequency bands [alpha (8 to 12 Hz), beta (13 to 30 Hz), and gamma (30 to < 50 Hz)], as connectivity in these bands has been previously reported as abnormal in SeLECTS in the resting state [9, 10, 11]. Second, since some components of language processing occur within tens to hundreds of milliseconds [5], we tested if group differences were better seen in more specific time windows. To do this, we calculated connectivity in 1 s time windows, beginning before noun onset and advancing through the first 2 s of the verb generation task. Third, we explored the impact of unbalanced variables, running models adjusting for SES and IQ [45]. Fourth, we examined the impact that extreme cases (children with the highest connectivity) had on our conclusions by excluding these cases.

## 4 Results

### 4.1 Subjects

Of the 68 children who consented, one did not participate in the EEG session and four were excluded due to poor data quality, yielding 31 participants with SeLECTS and 32 age and sex‐matched controls in the final sample. Three SeLECTS participants and two controls did not complete neuropsychological testing, but their EEG data was included in the connectivity analysis.

### 4.2 Demographic, Clinical, Neurocognitive, and Data Quality Comparison

There were no significant group differences in age, sex, or degree of right‐handedness (Table 1). Children with SeLECTS had lower socioeconomic status, IQ, and CTOPP‐2 Composite scores than controls. There were no group differences in task performance or EEG data quality measures (Table S1). Seventeen (55%) SeLECTS participants were taking ASM: 10 levetiracetam, seven oxcarbazepine.

**TABLE 1:** Demographics & cognitive scores for children with SeLECTS and controls.

Demographics	Group		
	SeLECTS (*n* = 31)	Controls (*n* = 32)	*p*
Age (years), mean, SD	9.7 ± 2.0	9.1 ± 2.0	0.22
Sex (male), *n* (%)	22 (71)	18 (56)	0.34
Medication, *n* (%)	17 (55)	N/A	N/A
Edinburgh Handedness Inventory^a^, mean, SD	0.82 ± 0.14	0.77 ± 0.17	0.25
Hollingshead SES Index, mean, SD	54.6 ± 11.1	60.3 ± 4.4	0.01
ADHD status, *n* (%)	5 (16)	6 (19)	0.73

Neuropsychological assessments	SeLECTS (*n* = 28)	Controls (*n* = 30)	*p*
WASI‐II IQ	100.3 ± 14.2	115.2 ± 14.8	0.0004
CTOPP‐2 Phonological Awareness Composite Score	102.3 ± 14.1	113.4 ± 13.4	0.003

Abbreviations: ADHD, attention deficit hyperactivity disorder; CTOPP‐2, Comprehensive Test of Phonological Processing‐2nd Edition; SeLECTS, self‐limited epilepsy with centrotemporal spikes; SES, socioeconomic status; WASI‐II IQ, Wechsler Abbreviated Scale of Intelligence‐2nd Edition, Intelligence Quotient.

^a^ Edinburgh Handedness Inventory (+1 = strongly right‐handed and −1 = left‐handed).

### 4.3 Group Differences in Connectivity

Across all regions and conditions, connectivity trended higher in the SeLECTS group compared to controls (Figure 2, Table S2).

**FIGURE 2:**
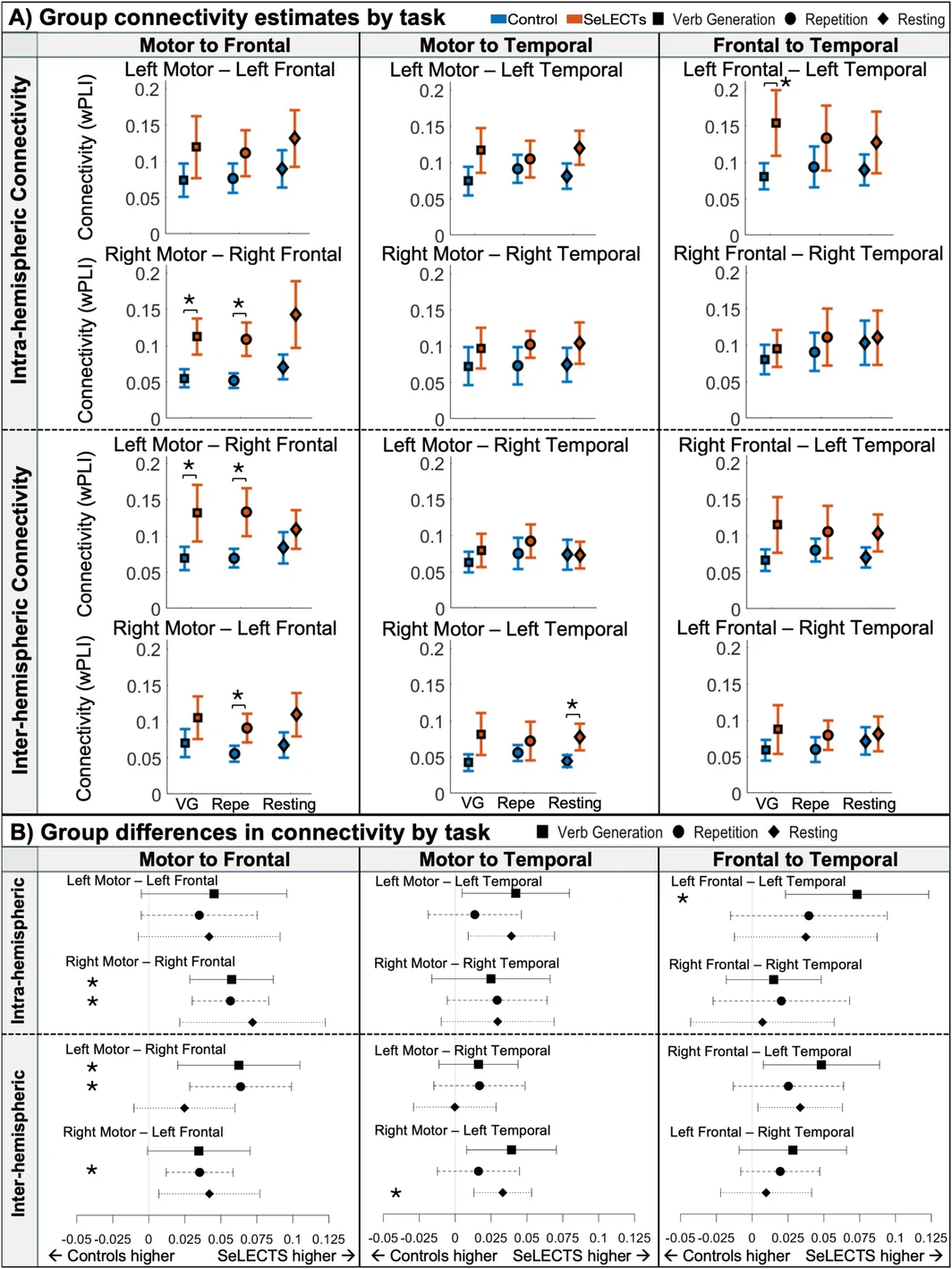
Group differences in theta‐band connectivity during all tasks. (A) Box and whisker plots represent estimated marginal mean connectivity with 95% confidence intervals for each group and task. (B) Forest plots represent differences in connectivity estimates between groups with 95% confidence intervals. *Indicate *p* < 0.0042 (adjusted significance threshold). Left column: Motor to inferior frontal connectivity; Middle column: Motor to superior temporal connectivity; Right column: Inferior frontal to superior temporal connectivity. Repe, repetition; VG, verb generation.

#### 4.3.1 Motor to IF Connectivity

SeLECTS children had significantly higher connectivity between the right IF region and both the left and right motor regions during the verb generation and repetition tasks. Connectivity was elevated between the left IF and right motor regions only during repetition.

#### 4.3.2 Motor to ST Connectivity

Connectivity was elevated in SeLECTS between the left ST and right motor regions during resting.

#### 4.3.3 IF to ST Connectivity

Connectivity between the left IF and left ST regions—the regions most implicated in language processing—was elevated in SeLECTS specifically during the verb generation task.

#### 4.3.4 Effect of ASM on Connectivity

When comparing three groups (SeLECTS+ASM, SeLECTS‐ASM, and controls), there were significant group differences in SES, IQ, and CTOPP‐2 scores (Table S3). Epilepsy severity did not differ between the SeLECTS+ASM and SeLECTS‐ASM groups (Table S4). Connectivity between the same region pairs as described above was significantly higher in the SeLECTS+ASM group than in controls (Figure S1, Table S5). In contrast, connectivity in the SeLECTS‐ASM group did not differ significantly from that in controls (Table S6). The SeLECTS+ASM group had higher connectivity than the SeLECTS‐ASM group in two regions (left IF to left ST regions during verb generation; left IF to right ST during repetition) (Table S7).

### 4.4 Association Between Connectivity and Phonological Awareness

#### 4.4.1 SeLECTS

High interhemispheric connectivity (connectivity between regions in opposite hemispheres) during the verb generation task, and to a lesser extent during the repetition task, was strongly associated with poor phonological awareness (Table 2, Figure 3). There were significant associations in five of the six interhemispheric region‐pairs during the verb generation task and three region‐pairs during the repetition task. Intra‐hemispheric connectivity (connectivity between regions in the same hemisphere) was not associated with phonological awareness. Resting connectivity was not associated with phonological awareness in SeLECTS. Associations remained significant in four of six interhemispheric pairs even after adjusting for SES, IQ, and handedness (see Section 4.5.3). When the patient with the lowest phonological awareness was excluded from the analyses, however, the associations between region‐pair connectivity and phonological awareness no longer met statistical significance (see Section 4.5.4).

**TABLE 2:** Association between clinical language performance and connectivity in children with SeLECTS.

		Region pair	Verb generation		Repetition		Resting	
			Estimate (95% Cl)	*p*	Estimate (95% Cl)	*p*	Estimate (95% Cl)	*p*
Motor to frontal	Intrahemispheric	LMotor–LFront	−16.8 (−51.3, 17.8)	0.34	−30.1 (−72.6, 12.4)	0.17	−50.1 (−89.8, −10.4)	0.01
		RMotor–RFront	−37.0 (−104.2, 30.2)	0.28	−73.1 (−128.5, −17.8)	0.01	−19.1 (−42.0, 3.9)	0.10
	Interhemispheric	LMotor–RFront	−65.7 (−106.4, −24.9)	**0.002**	−57.9 (−91.5, −24.4)	**0.0007**	−16.5 (−77.4, 44.5)	0.60
		RMotor–LFront	−41.9 (−95.4, 11.7)	0.13	−109.9 (−207.4, −12.5)	0.03	−33.9 (−67.3, −0.4)	0.05
Motor to temporal	Intrahemispheric	LMotor–LTemp	−19.7 (−76.4, 37.0)	0.50	−2.9 (−80.3, 74.5)	0.94	1.7 (−62.5, 66.0)	0.96
		RMotor–RTemp	−9.4 (−81.5, 62.7)	0.80	−33.0 (−124.9, 58.9)	0.48	9.0 (−49.9, 67.8)	0.77
	Interhemispheric	LMotor–RTemp	−61.3 (−98.6, −24.0)	**0.001**	−48.3 (−103.6, 6.9)	0.10	−67.0 (−132.4, −1.6)	0.05
		RMotor–LTemp	−55.8 (−81.5, −30.1)	**0.0001**	−46.8 (−71.0, −22.6)	**0.0001**	−48.2 (−119.9, 23.5)	0.19
Frontal to temporal	Intrahemispheric	LFront–LTemp	−33.1 (−60.3, −5.9)	0.02	−29.4 (−50.6, −8.1)	0.007	−23.7 (−50.1, 2.8)	0.08
		RFront–RTemp	−34.2 (−79.2, 10.8)	0.14	−41.2 (−61.4, −20.9)	**0.0001**	−26.3 (−44.5, −8.1)	0.005
	Interhemispheric	RFront–LTemp	−53.6 (−75.1, −32.0)	**0.0001**	−42.9 (−60.1, −25.7)	**0.0001**	−0.008 (−63.1, 63.1)	0.99
		LFront–RTemp	−40.3 (−66.6, −14.0)	**0.003**	−56.8 (−117.6, 4.0)	0.08	−77.4 (−142.2, −12.5)	0.02

*Note:* *p*‐values meeting threshold (*p* < 0.0042) are in bold.

Abbreviations: LFront, left inferior frontal; LMotor, left motor; LTemp, left superior temporal; RFront, right inferior frontal; RMotor, right motor; RTemp, right superior temporal.

**FIGURE 3:**
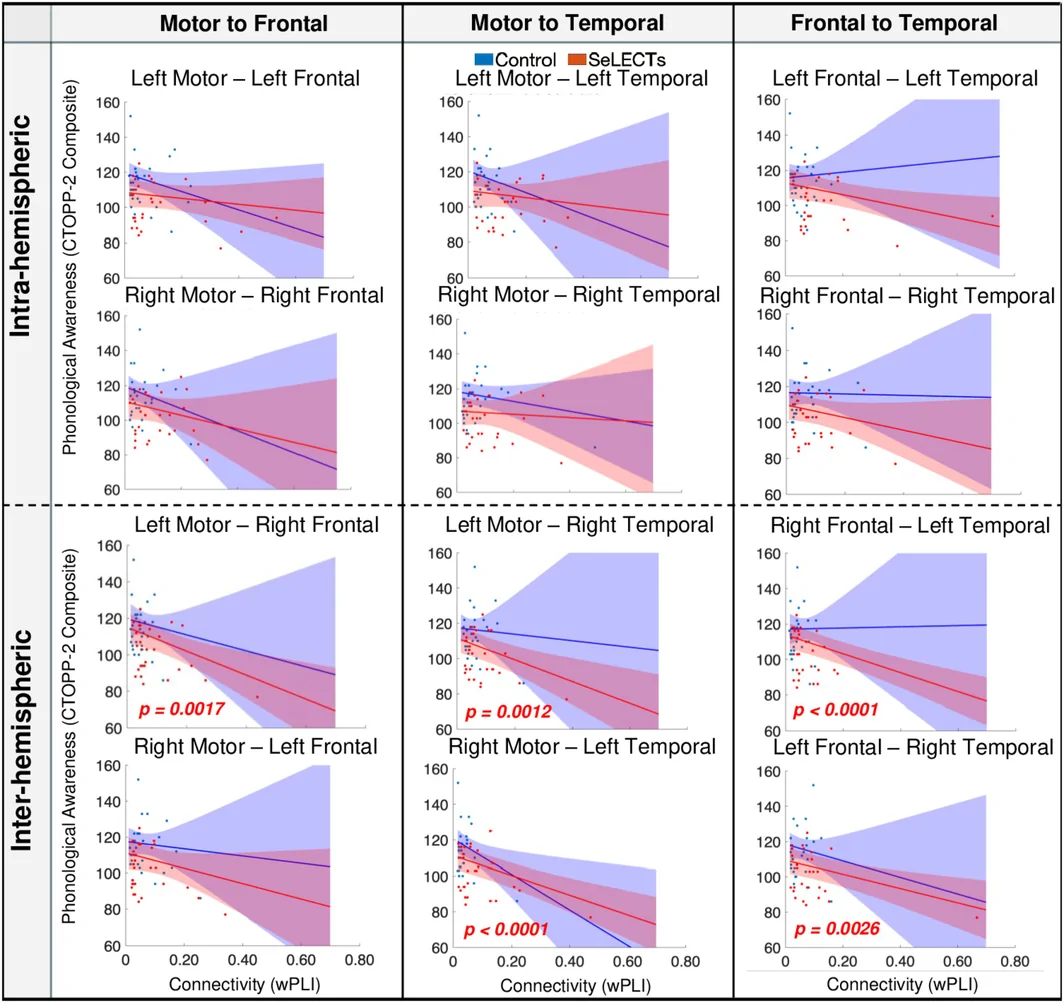
Association between connectivity and clinical language performance in children with SeLECTS and controls during the verb generation task (Exploratory analysis). Scatter plots show mean connectivity vs. CTOPP‐2 Composite scores with estimated marginal fit lines and 95% confidence intervals. *P*‐values meeting threshold (*p* < 0.0042) noted and color coded to group (red = SeLECTS; blue = controls). Left column: Motor to Inferior Frontal connectivity; Middle column: Motor to Superior Temporal connectivity; Right column: Inferior Frontal to Superior Temporal connectivity. Sensitivity analyses excluding one influential case with extreme connectivity values attenuated these associations (Figure S3, Table S15).

#### 4.4.2 Controls

Higher right motor to right ST connectivity in resting was associated with lower phonological awareness. There were no other significant associations in controls (Table 3, Figure 3). Associations were significant in three interhemispheric pairs after adjusting for SES, IQ, and handedness (see Section 4.5.3).

**TABLE 3:** Association between clinical language performance and connectivity in controls.

		Region pair	Verb Generation		Repetition		Resting	
			Estimate (95% Cl)	*p*	Estimate (95% Cl)	*p*	Estimate (95% Cl)	*p*
Motor to Frontal	Intrahemispheric	LMotor–LFront	−52.5 (−119.4, 14.3)	0.12	−29.3 (−102.6, 44.1)	0.43	−44.3 (−103.8, 15.3)	0.15
		RMotor–RFront	−60.8 (−173.7, 52.1)	0.29	−95.9 (−252.2, 60.4)	0.23	−76.9 (−163.3, 9.5)	0.08
	Interhemispheric	LMotor–RFront	−42.2 (−145.7, 61.3)	0.42	−31.7 (−137.6, 74.2)	0.56	−15.8 (−77.9, 46.3)	0.62
		RMotor–LFront	−19.5 (−114.6, 75.6)	0.69	−128.5 (−233.0, −23.9)	0.02	−80.9 (−163.4, 1.5)	0.05
Motor to temporal	Intrahemispheric	LMotor–LTemp	−60.5 (−183.6, 62.6)	0.34	−61.6 (−132.6, 9.4)	0.09	39.9 (−23.3, 103.2)	0.22
		RMotor–RTemp	−28.1 (−80.0, 23.9)	0.29	−31.2 (−87.6, 25.1)	0.28	−58.2 (−87.5, −28.9)	**0.0001**
	Interhemispheric	LMotor–RTemp	−17.9 (−164.7, 129.0)	0.81	−66.6 (−113.9, −19.4)	0.006	−60.9 (−122.6, 0.8)	0.05
		RMotor–LTemp	−98.2 (−179.4, −17.0)	0.02	−135.2 (−242.1, −28.3)	0.01	−18.8 (−232.7, 195.0)	0.86
Frontal to temporal	Intrahemispheric	LFront–LTemp	18.2 (−78.7, 115.2)	0.71	2.3 (−41.1, 45.6)	0.92	10.2 (−49.5, 69.9)	0.74
		RFront–RTemp	−1.5 (−83.2, 80.1)	0.97	−47.5 (−95.3, 0.32)	0.05	−51.8 (−89.0, −14.7)	0.006
	Interhemispheric	RFront–LTemp	4.1 (−125.0, 133.1)	0.95	−12.3 (−90.6, 65.9)	0.76	16.8 (−59.7, 93.2)	0.67
		LFront–RTemp	−47.2 (−143.9, 49.4)	0.34	−93.2 (−162.2, −24.2)	0.008	−76.2 (−142.9, −9.4)	0.03

*Note:* *p*‐values meeting threshold (*p* < 0.0042) are in bold.

Abbreviations: LFront, left inferior frontal; LMotor, left motor; LTemp, left superior temporal; RFront, right inferior frontal; RMotor, right motor; RTemp, right superior temporal.

### 4.5 Supplementary Analyses

#### 4.5.1 Connectivity in Other Frequency Bands

There were no significant group differences in the alpha, beta, or gamma bands (Tables S8–S10).

#### 4.5.2 Connectivity Over Specific Time Windows

Baseline theta connectivity before noun onset did not differ between groups (Figure S2). The SeLECTS group had higher connectivity than controls between the bilateral motor and bilateral IF and between bilateral motor and left ST, most prominently from 0.5 to 1.5 s after noun onset. Left IF to left ST connectivity was also elevated in SeLECTS in this period. Results were consistent with analyses using longer 3 s epochs.

#### 4.5.3 Connectivity When Adjusting for IQ & SES

Adjusting for IQ and SES showed only a mild attenuation of the original group difference results (Table S11). While some regions (right motor and left frontal during repetition; right motor and left ST during resting) were no longer statistically significant after multiple comparison correction, the results are largely consistent with original group analyses (e.g., left IF to left ST regions during verb generation; right IF to motor regions during verb generation and repetition). After adjusting for SES, IQ, and handedness, associations between connectivity and phonological awareness remained significant in four of six interhemispheric pairs in SeLECTS (Table S12) and were seen in three interhemispheric pairs in controls (Table S13).

#### 4.5.4 Connectivity When Excluding Potential Outliers

Sensitivity analyses examined the influence of individual cases on group differences in connectivity and connectivity‐cognition associations (Figures S3–S5). These identified that one participant with high connectivity (> 95th percentile) and the lowest language score in the SeLECTS group showed high statistical leverage. When this case was excluded, group differences in connectivity remained robust across most previously identified region‐to‐region pairs (Table S14). Connectivity‐cognition associations no longer met the significance threshold (Table S15), though the pattern of stronger associations between phonological awareness and interhemispheric connectivity measures persisted. This participant had the highest spike burden during the research EEG of any participant, suggesting this may represent an extreme phenotype. Since the connectivity‐cognition relationships in our sample are influenced by individual extreme cases, however, these results require replication in larger samples, including more children with frequent spikes.

## 5 Discussion

We investigated whether language problems associated with epilepsy disrupt functional connectivity. Using high‐density EEG recordings, we measured connectivity between the motor cortices—the source of interictal spikes and seizures in SeLECTS—and IF and ST language regions. We found three key patterns. First, while performing a language task, children with SeLECTS have significantly higher connectivity between motor and IF regions than controls, with the largest differences involving the *right* IF region. Second, contrary to our hypothesis, connectivity between IF and ST language regions in the left hemisphere was also *elevated* in SeLECTS. Third, higher interhemispheric connectivity was strongly associated with worse phonological awareness in children with SeLECTS, suggesting that excessive connectivity impedes language processing. Our findings suggest hyperconnectivity contributes to language difficulties in SeLECTS, offering insights into therapeutic targets.

### 5.1 Motor to Inferior Frontal Connectivity Differences

Motor‐to‐IF connectivity is elevated in SeLECTS specifically during language tasks but not during resting. We were surprised that differences were task specific as we hypothesized that motor cortex hyperconnectivity would be driven by spikes and hence present regardless of cognitive demands. Given that motor‐to‐IF hyperconnectivity is observed during both verb generation and repetition tasks, we speculate it is elicited by a shared cognitive component—such as attention, working memory, or articulatory planning [18]—rather than a semantic retrieval component unique to verb generation. The predominance of right IF findings is particularly notable, as this region has been implicated in inhibitory control [46], a key component for attentional processes which are disrupted in SeLECTS [47]. Supporting this, a spike‐timed EEG‐fMRI study [48] found that centrotemporal spikes activate both the right IF and the caudate nucleus, structures important for attention regulation. Alternatively, the right IF region may play a more prominent role in expressive language in SeLECTS. Verb generation elicits excessive right frontal activation both in children with active SeLECTS [2, 49] and in adolescents with a history of the disorder [50]. Lateralization of the anterior language network is atypical in various focal epilepsies [51] and may be specifically disrupted by SeLECTS pathology [49], requiring affected children to recruit additional right hemispheric regions to maintain normal task performance.

### 5.2 Motor to Superior Temporal Connectivity Differences

Motor‐to‐ST connectivity was similar between groups, with the only difference being elevated right motor to left ST connectivity during resting but not during tasks. Prior studies have found that SeLECTS disrupts the default mode network (DMN), a set of interconnected brain regions that coactivate during the resting state. DMN disruption affects cognitive efficiency by interfering with resource allocation between task‐positive and task‐negative networks, contributing to language difficulties [52]. Resting connectivity and DMN dysregulation may represent another mechanism through which motor‐cortex spikes impact distributed cognitive networks, including those supporting language processing.

### 5.3 Frontal to Temporal Connectivity Differences

Contrary to our hypothesis and previous neuroimaging studies showing reduced structural [33] and resting‐state functional [53] connectivity in the left hemisphere language network, we found increased left IF to left ST connectivity in SeLECTS specifically during the verb generation task. This suggests that language network connectivity during semantic retrieval is affected in SeLECTS. One explanation is that elevated connectivity reflects delayed maturation of the language network. This interpretation aligns with fMRI studies showing that activation in higher‐level language regions decreases with age as language processing becomes more efficient [6] and increases again (even in adulthood) when tasks become more semantically challenging [54]. Children with SeLECTS may require more intensive utilization of language regions to achieve the same behavioral output as typically developing children, resulting in higher measured connectivity. Supporting this interpretation, task performance did not differ between groups despite these neurophysiological differences, suggesting compensatory mechanisms. A second interpretation is that higher connectivity represents accelerated language network development; one MEG study found that theta band connectivity increases over time in children and is associated with improving language function [20]. We think this is unlikely in SeLECTS, where higher connectivity is associated with poorer phonological awareness. A third explanation is that SeLECTS induces atypical development of the language network, much as seizures and spikes can alter connectivity in animal models. Adolescents with a history of resolved SeLECTS still have different EEG activity during verb generation, indicating lasting network reorganization [50]. The critical distinction between delayed versus atypical development has significant implications for prognosis and intervention. With delayed development, connectivity abnormalities may normalize with age or seizure resolution whereas atypical development demands targeted early interventions. Longitudinal studies examining how connectivity patterns evolve during active epilepsy and after resolution, and the relationship between connectivity and language, will be crucial to address this question.

### 5.4 Effect of ASMs

ASM effects vary by region. Motor‐to‐IF connectivity follows a gradient: controls have lowest connectivity, unmedicated children with SeLECTS have mid‐level connectivity, and medicated children with SeLECTS have highest connectivity. Connectivity in unmedicated children with SeLECTS more closely resembles that of controls than that of medicated children, but small sample size limits power. Interestingly, left IF‐to‐ST connectivity is significantly higher in children taking ASMs than either controls or unmedicated children. One potential explanation is that more severe epilepsy is associated with both higher connectivity and ASM prescription. However, traditional epilepsy severity measures did not significantly differ between ASM‐ and ASM+ groups (Table S7), suggesting that the observed connectivity differences do not simply reflect seizure burden. This raises the possibility that ASMs directly increase connectivity, but prior literature is mixed. When followed over 2 years, EEG functional connectivity remained stable in children with SeLECTS who started an ASM but increased in those who remained unmedicated [55]. In contrast, a MEG study found an increase in functional connectivity in the default mode network after initiation of an ASM [56]. Both studies support ASMs' ability to alter connectivity in SeLECTS but an understanding of how and whether they normalize connectivity is limited given the lack of a control group. A cross‐sectional fMRI study also found that both medicated and unmedicated children with SeLECTS have different functional connectivity than controls, but it was reduced in children on ASMs and *elevated* in children off ASMs [57]. Disentangling the effects of epilepsy severity vs. ASMs on connectivity is important as there is still equipoise regarding the decision to start ASMs. Prospective studies examining connectivity before and after ASM initiation are required to understand the causal role that ASMs play in determining connectivity and cognition.

### 5.5 Association Between Connectivity and Phonological Awareness

Our exploratory analyses suggest that higher interhemispheric connectivity during the verb generation and repetition tasks is associated with worse phonological awareness in children with SeLECTS and not in controls. Two fMRI studies also found that interhemispheric connectivity is associated with language abilities in SeLECTS. One highly concordant study [15] noted that higher motor‐to‐left‐temporal connectivity was associated with lower verbal and full‐scale IQ in SeLECTS. In contrast, the second study found that higher left motor to right frontal connectivity was associated with *better* language scores [2]. While the direction of the association varies, both suggest that interhemispheric connectivity plays a role in language function. Notably, both studies looked at connectivity during the resting state, whereas we focused on a language task. Children with dyslexia (which also impairs phonological awareness) [58] also have greater interhemispheric connectivity than controls during a word‐tracking task and high connectivity is associated with poor phonological awareness. We propose that high interhemispheric connectivity is associated with poor language performance because it signifies that a segregated left‐lateralized language network has not (yet) developed. Corroborating this, appropriate activation of limited, task‐appropriate brain networks (rather than wider brain regions) is associated with better cognitive performance in SeLECTS [52, 59] and other focal epilepsies [60]. Whether excessive connectivity is a pathology that directly interferes with language function vs. a compensatory response to underlying network dysfunction could be tested using noninvasive neurostimulation. Critically, however, sensitivity analyses revealed language‐connectivity associations are influenced by an individual case with high connectivity, poor language, and high spike burden. This case theoretically represents an extreme phenotype consistent with our hypothesis, but replication of the connectivity‐language relationship is needed.

### 5.6 Limitations

First, while the group connectivity differences were pronounced, clear task‐specific differences within groups were minimal, suggesting that the connectivity measure was not sensitive to differences in brain activity elicited by verb generation and repetition. We tested whether using shorter time windows that better approximate typical language processing better captured task‐induced differences (see Section 4.5). With this sliding‐window approach, significant group connectivity differences were again present during the task but not at baseline; within‐group changes in connectivity over the task could be seen in SeLECTS but not controls, and were not significant in either group. We suspect that the larger connectivity changes in SeLECTS are due to the task being more challenging for this group. Future studies should test whether varying task difficulty or varying the comparison (e.g., using a nonword repetition task to uncouple phonological awareness from vocabulary knowledge) more consistently identifies language‐specific neural processes. Second, our tasks did not capture natural language complexity, and future studies measuring naturalistic language abilities would enhance generalizability. Third, our scalp‐based connectivity measurements lack spatial specificity. While source localization would have improved anatomical precision, this approach was difficult due to the lack of individual neuroimaging data. Nevertheless, we observed clear regional differences in connectivity that varied with task demands in sensor space, suggesting that our approach captured meaningful functional distinctions between regions. Fourth, our groups were not perfectly matched for all demographic or cognitive variables. IQ is particularly interesting, because it is moderately associated with connectivity and significantly associated with SeLECTS status. While theoretically IQ could confound the differences in connectivity observed between groups, models adjusting for IQ do not substantially change our conclusions. IQ is also associated with phonological awareness, but we believe it is more likely to be on the causal pathway between connectivity and phonological awareness than a confounder that is independently associated with these two variables [61]. Critically, our supplementary analyses adjusting for SES and IQ did not alter the negative relationship between high connectivity and phonological awareness. Future studies with better matching would strengthen conclusions about the causal association between connectivity and language performance. Fifth, different ASMs may have different effects on connectivity, and we lack the power to reliably explore these differences. Sixth, our connectivity‐cognition findings require cautious interpretation given the sensitivity to an influential case. The case does represent a legitimate extreme phenotype consistent within our theoretical framework, and our modest sample size provides limited power to reliably detect small‐to‐medium effect sizes. Definitive conclusions about connectivity‐cognition relationships await replication in larger samples. In contrast, our primary findings regarding group differences in connectivity showed robustness to sensitivity analyses.

## 6 Conclusion

Children with SeLECTS have aberrant hyperconnectivity between epileptic motor and language cortices that arises specifically during language processing and is strongly associated with phonological processing deficits. Our study provides support for suppressing hyperconnected motor‐language pathways, and particularly interhemispheric connections, to normalize network function and improve language outcomes in children with SeLECTS, establishing a foundation for precision neuromodulation approaches in pediatric epilepsy.

## Author Contributions

W.Q.: conceptualization, data curation, investigation, formal analysis, visualization, software, writing – original draft, writing – review and editing. K.L.: conceptualization, data curation, investigation, formal analysis, visualization, software, writing – original draft, writing – review and editing. K.C.N.: methodology, software, data curation, validation, writing – review and editing. M.M.: data curation, validation, writing – review and editing. X.S.: software, validation, writing – review and editing. L.S.M.: formal analysis, writing – review and editing. W.W.: methodology, software, writing – review and editing. Z.H.: methodology, writing – review and editing. F.M.B.: conceptualization, methodology, software, data curation, investigation, validation, formal analysis, supervision, visualization, writing – original draft, writing – review and editing, project administration, resources, funding acquisition.

## Funding

Funding was provided by K23 NS116110, the Doris Duke Foundation and the Rita Allen Foundation, a gift from the Principe & O'Farrell family, and the Wu Tsai Neurosciences Institute to F.M.B. Funding was provided by the Maternal & Child Health Research Institute to X.S.

## Conflicts of Interest

The authors declare no conflicts of interest.

## Supporting information


**Table S1:** Preprocessing and task performance results for children with SeLECTS and Controls.
**Table S2:** Group differences in connectivity for children with SeLECTS and Controls during all tasks.
**Table S3:** Subject demographics by medication usage.
**Table S4:** Epilepsy severity for children with SeLECTS.
**Table S5:** Group differences in connectivity for children with SeLECTS on ASM and Controls during all tasks.
**Table S6:** Group differences in connectivity for children with SeLECTS off ASM and Controls during all tasks.
**Table S7:** Group differences in connectivity for children with SeLECTS on and off ASM during all tasks.
**Table S8:** Group differences in connectivity for children with SeLECTS and Controls in ALPHA band during all tasks.
**Table S9:** Groups differences in connectivity for children with SeLECTS and Controls in BETA band during all tasks.
**Table S10:** Group differences in connectivity for children with SeLECTS and Controls in GAMMA band during all tasks.
**Table S11:** Group differences in connectivity for children with SeLECTS and Controls during all tasks adjusting for IQ and SES.
**Table S12:** Association between clinical language performance and connectivity in children with SeLECTS adjusting for age, sex, socioeconomic status, IQ, and handedness.
**Table S13:** Association between clinical language performance and connectivity in Controls adjusting for age, sex, socioeconomic status, IQ, and handedness.
**Table S14:** Group differences in connectivity for children with SeLECTS and Controls during all tasks without potential outlier.
**Table S15:** Association between clinical language performance and connectivity in children with SeLECTS without potential outlier.
**Figure S1:** Impact of anti‐seizure medication (ASM) use on connectivity.
**Figure S2:** Group differences in connectivity during the verb generation task with sliding time window analysis.
**Figure S3:** Association between clinical language performance and connectivity in children with SeLECTS and Controls during the verb generation task with marked influential case.
**Figure S4:** Association between clinical language performance and connectivity in children with SeLECTS and Controls during the repetition task with marked influential case.
**Figure S5:** Association between clinical language performance and connectivity in children with SeLECTS and Controls during the resting task with marked influential case.
**Methods S1:** Epilepsy severity for children with SeLECTS.
**Methods S2:** Group differences in connectivity during the verb generation task with sliding time window analysis.

## Data Availability

The datasets generated and analyzed in the study are not currently publicly available due to privacy concerns with de‐identification of clinical data. However, the full dataset is available from the corresponding author by request.
