# Supplementary material for: Elevated Connectivity During Language Processing Is Associated With Cognitive Performance in SeLECTS
Source: Ann Clin Transl Neurol. 2026 Apr 21;13(11):2309–22. doi: 10.1002/acn3.70369 (PMC13394123; doi:10.1002/acn3.70369)
Supplement: Supplementary file 1 [file ACN3-13-2309-s001.docx]

**Supplementary Table 1.** Preprocessing and task performance results for children with SeLECTS and Controls.

| **Data Quality** | **Group** | |  |  |
| --- | --- | --- | --- | --- |
|  | SeLECTS (n=31) | Controls (n=32) | t-statistic | p-value |
| Number of rejected channels | *5.6 +/- 2.0* | *5.4 +/- 2.2* | *0.38* | *0.70* |
| % of rejected ICs | *23.4% +/- 11.1%* | *20.4% +/- 5.2%* | *1.4* | *0.18* |
| **Number of Epochs** |  |  |  |  |
| Verb Generation Task | *47.5 +/- 6.3* | *49.3 +/- 2.0* | *1.6* | *0.12* |
| Repetition Task | *40.4 +/- 9.3* | *43.0 +/- 7.9* | *1.2* | *0.24* |
| Resting Task | *50 +/- 0* | *49.7 +/- 1.6* | *1.1* | *0.28* |
| **Task Response** |  |  |  |  |
| Verb Response | *87.4 +/- 26.9* | *88.6 +/- 33.7* | *0.15* | *0.88* |
| Task Error | *3.2 +/- 9.0* | *1.1 +/- 1.5* | *1.2* | *0.24* |
| Retrieval Error | *23.5 +/- 17.8* | *32.3 +/- 22.7* | *1.7* | *0.1* |

*ICs = Independent components

**Supplementary Table 2.** Group differences in connectivity for children with SeLECTS and Controls during all tasks.

|  |  | Region Pair | Verb Generation | | Repetition | | Resting | |
| --- | --- | --- | --- | --- | --- | --- | --- | --- |
|  |  |  | Estimate  (95% Cl) | p-value | Estimate (95% Cl) | p-value | Estimate (95% Cl) | p-value |
| Motor to Frontal | Intra-  hemispheric | LMotor — LFront | *0.05 (-0.005-0.1)* | *0.08* | *0.04  (-0.005-0.08)* | *0.09* | *0.04  (-0.007-0.09)* | *0.10* |
|  |  | RMotor — RFront | *0.06  (0.03-0.09)* | ***0.0001*** | *0.06  (0.03-0.08)* | ***0.0001*** | *0.07  (0.02-0.1)* | *0.005* |
|  | Inter-  hemispheric | LMotor — RFront | *0.06  (0.02-0.1)* | ***0.004*** | *0.06  (0.03-0.1)* | ***0.0004*** | *0.02  (-0.01-0.06)* | *0.17* |
|  |  | RMotor — LFront | *0.03  (-0.0009-0.07)* | *0.06* | *0.04  (0.01-0.06)* | ***0.003*** | *0.04  (0.007-0.08)* | *0.02* |
| Motor to Temporal | Intra-  hemispheric | LMotor — LTemp | *0.04  (0.005-0.08)* | *0.03* | *0.01  (-0.02-0.05)* | *0.41* | *0.04  (0.009-0.07)* | *0.01* |
|  |  | RMotor — RTemp | *0.02  (-0.02-0.07)* | *0.24* | *0.03  (-0.006-0.06)* | *0.10* | *0.03  (-0.01-0.07)* | *0.14* |
|  | Inter-  hemispheric | LMotor — RTemp | *0.02  (-0.01-0.04)* | *0.25* | *0.02  (-0.01-0.05)* | *0.30* | *-0.0002  (-0.03-0.03)* | *0.99* |
|  |  | RMotor — LTemp | *0.04  (0.008-0.07)* | *0.01* | *0.02  (-0.01-0.04)* | *0.27* | *0.03  (0.01-0.05)* | ***0.001*** |
| Frontal to Temporal | Intra-  hemispheric | LFront — LTemp | *0.07  (0.02-0.12)* | ***0.004*** | *0.04  (-0.02-0.09)* | *0.16* | *0.04  (-0.01-0.09)* | *0.14* |
|  |  | RFront — RTemp | *0.02  (-0.02-0.05)* | *0.37* | *0.02  (-0.03-0.07)* | *0.40* | *0.007  (-0.04-0.06)* | *0.78* |
|  | Inter-  hemispheric | RFront — LTemp | *0.05  (0.008-0.09)* | *0.02* | *0.03  (-0.01-0.06)* | *0.20* | *0.03  (0.004-0.06)* | *0.03* |
|  |  | LFront — RTemp | *0.03 (-0.009-0.07)* | *0.14* | *0.02  (-0.008-0.05)* | *0.16* | *0.01  (-0.02-0.04)* | *0.55* |

P-values meeting threshold (p<0.0042) are in bold. LFront: Left Inferior Frontal; LTemp: Left Superior Temporal; LMotor: Left Motor; RFront: Right Inferior Frontal; RTemp: Right Superior Temporal; RMotor: Right Motor.

**Supplementary Table 3.** Subject demographics by medication usage

|  | **Group** | | | |
| --- | --- | --- | --- | --- |
| **Demographics** | SeLECTS-ASM (n=14) | SeLECTS+ASM (n=17) | Controls (n=32) | p-value |
| Age (years) mean, SD | *10.2 +/- 2.0* | *9.3 +/- 1.9* | *9.1 +/- 2.0* | *0.20* |
| Sex (male), n (%) | *10 (71)* | *12 (71)* | *18 (56)* | *0.48* |
| Edinburgh Handedness Inventory* | *0.83 +/- 0.09* | *0.80+/- 0.17* | *0.77 +/- 0.17* | *0.45* |
| Hollingshead SES Index | *56.3 +/- 9.8* | *53.2 +/- 12.4* | *60.3 +/- 4.4* | *0.03* |
| **Neuropsychological Testing** | SeLECTS-ASM (n=12) | SeLECTS+ASM (n=16) | Controls (n=30) | p-value |
| WASI-II IQ | *105.7 +/- 13.6* | *97+/- 14.0* | *115.2 +/- 14.8* | *0.0006* |
| CTOPP-2 Phonological Awareness Score | *107.6 +/- 13.7* | *98.4 +/- 13.4* | *113.4 +/- 13.4* | *0.003* |

*Edinburgh Handedness Inventory (+1 = strongly right-handed and -1 = left-handed); ADHD: Attention Deficit Hyperactivity Disorder; CTOPP-2: Comprehensive Test of Phonological Processing-2^nd^ Edition; SeLECTS-ASM: SeLECTS children not taking a daily antiseizure medication; SeLECTS+ASM: SeLECTS children taking a daily antiseizure medication; SES: Socioeconomic Status; WASI-II IQ: Wechsler Abbreviated Scale of Intelligence-2^nd^ Edition Intelligence Quotient.

**Supplementary Methods & Table 4.** Epilepsy severity for children with SeLECTS.

# **Assessment of Epilepsy Severity:** There is not a validated scale of epilepsy severity for SeLECTS. The Global assessment of the severity of epilepsy (GASE) is a scale validated for assessing epilepsy severity in children that is filled out by the patient’s treating physician. We chose questions from this assessment that were relevant to SeLECTS and could be extracted from the medical record to compare epilepsy between children with SeLECTS taking vs. not taking daily prophylactic ASMs. We compared groups using the t-test and chi-squared test for continuous and categorical values.

|  | SeLECTS - ASM  (n=14) | SeLECTS + ASM  (n=17) | test statistic | p-value |
| --- | --- | --- | --- | --- |
| Number of Lifetime Seizures | *7.3 +/- 10.3* | *12.9 +/- 12.8* | *1.3* | *0.21* |
| Hx Seizures > 5min, n (%) | *2 (14)* | *2 (12)* | *0.11* | *0.74* |
| Seizure state |  |  |  |  |
| *Asleep Only* | *9 (64)* | *6 (35)* | *1.6* | *0.21* |
| *Awake & Asleep* | *5 (36)* | *11 (65)* | *1.6* | *0.21* |
| Hx Seizures with Secondary Generalization, n (%) | *10 (71)* | *12 (71)* | *0.12* | *0.73* |

ASM: Antiseizure medication; Hx: history; SeLECTS-ASM: SeLECTS children not taking a daily antiseizure medication; SeLECTS+ASM: SeLECTS children taking a daily antiseizure medication.

**Conclusion:** Children with SeLECTS taking a daily antiseizure medication had a higher number of lifetime seizures, more seizures when both awake and asleep, and a history of seizures with more secondary generalization, though none of these differences were significant between groups.

**Supplementary Figure 1.** Impact of antiseizure medication (ASM) use on connectivity.

 Plots represent estimated marginal mean connectivity with 95% confidence interval for each group and task. * indicate p < 0.0042 (adjusted significance threshold). Left column: Motor to Inferior Frontal connectivity; Middle column: Motor to Superior Temporal connectivity; Right column: Inferior Frontal to Superior Temporal connectivity. VG = Verb Generation; Repe = Repetition.

**Supplementary Table 5.** Group differences in connectivity for Children with SeLECTS on ASM (SeLECTS+ASM) and Controls during all tasks.

|  |  | Region Pair | Verb generation | | Repetition | | Resting | |
| --- | --- | --- | --- | --- | --- | --- | --- | --- |
|  |  |  | Estimate  (95% Cl) | p-value | Estimate  (95% Cl) | p-value | Estimate  (95% Cl) | p-value |
| Motor to Frontal | Intra-hemispheric | LMotor — LFront | *0.08 (0.01, 0.16)* | *0.02* | *0.06 (0.004, 0.11)* | *0.04* | *0.08 (0.01, 0.14)* | *0.02* |
|  |  | RMotor — RFront | *0.08 (0.04, 0,12)* | ***0.0001*** | *0.08 (0.04, 0.11)* | ***0.0001*** | *0.09 (0.02, 0.17)* | *0.02* |
|  | Inter-hemispheric | LMotor — RFront | *0.08 (0.03, 0.12)* | ***0.001*** | *0.09 (0.04, 0.13)* | ***0.0003*** | *0.03 (-0.009, 0.08)* | *0.12* |
|  |  | RMotor — LFront | *0.06 (0.006, 0.11)* | *0.03* | *0.05 (0.02, 0.08)* | ***0.0004*** | *0.06 (0.02, 0.11)* | *0.009* |
| Motor to Temporal | Intra-hemispheric | LMotor — LTemp | *0.07 (0.01, 0.12)* | *0.02* | *0.03 (-0.01, 0.07)* | *0.16* | *0.05 (0.01, 0.08)* | *0.008* |
|  |  | RMotor—RTemp | *0.02 (-0.03, 0.07)* | *0.42* | *0.03 (-0.006, 0.07)* | *0.10* | *0.03 (-0.01, 0.08)* | *0.13* |
|  | Inter-hemispheric | LMotor — RTemp | *0.03 (-0.01, 0.07)* | *0.16* | *0.03 (-0.01, 0.07)* | *0.15* | *0.01 (-0.02, 0.05)* | *0.54* |
|  |  | RMotor — LTemp | *0.07 (0.01, 0.12)* | *0.01* | *0.03 (-0.02, 0.08)* | *0.25* | *0.04 (0.01, 0.07)* | ***0.003*** |
| Frontal to Temporal | Intra-hemispheric | LFront — LTemp | *0.13 (0.05, 0.2)* | ***0.0009*** | *0.08 (0.0003, 0.16)* | *0.05* | *0.05 (-0.01, 0.12)* | *0.11* |
|  |  | RFront — RTemp | *0.03 (-0.02, 0.08)* | *0.21* | *0.06 (-0.009, 0.13)* | *0.09* | *0.03 (-0.04, 0.10)* | *0.35* |
|  | Inter-hemispheric | RFront — LTemp | *0.08 (0.02, 0.14)* | *0.01* | *0.05 (-0.01, 0.11)* | *0.13* | *0.03 (-0.0008, 0.07)* | *0.06* |
|  |  | LFront — RTemp | *0.05 (-0.005, 0.11)* | *0.07* | *0.04 (0.007, 0.08)* | *0.02* | *0.04 (-0.006, 0.08)* | *0.10* |

P-values meeting threshold (p<0.0042) are in bold. LFront: Left Inferior Frontal; LTemp: Left Superior Temporal; LMotor: Left Motor; RFront: Right Inferior Frontal; RTemp: Right Superior Temporal; RMotor: Right Motor.

**Supplementary Table 6.** Group differences in connectivity for Children with SeLECTS off ASM (SeLECTS-ASM) and Controls during all tasks.

|  |  | Region Pair | Verb generation | | Repetition | | Resting | |
| --- | --- | --- | --- | --- | --- | --- | --- | --- |
|  |  |  | Estimate  (95% Cl) | p-value | Estimate  (95% Cl) | p-value | Estimate  (95% Cl) | p-value |
| Motor to Frontal | Intra-hemispheric | LMotor — LFront | *-0.006 (-0.05, 0.03)* | *0.77* | *0.0009 (-0.03, 0.04)* | *0.96* | *-0.005 (-0.05, 0.04)* | *0.86* |
|  |  | RMotor — RFront | *0.03 (-0.001, 0.06)* | *0.06* | *0.03 (-0.007, 0.06)* | *0.12* | *0.04 (-0.003, 0.09)* | *0.07* |
|  | Inter-hemispheric | LMotor — RFront | *0.04 (-0.03, 0.11)* | *0.24* | *0.03 (-0.02, 0.08)* | *0.18* | *0.01 (-0.03, 0.06)* | *0.61* |
|  |  | RMotor — LFront | *0.007 (-0.03, 0.04)* | *0.68* | *0.009 (-0.02, 0.03)* | *0.48* | *0.01 (-0.03, 0.05)* | *0.52* |
| Motor to Temporal | Intra-hemispheric | LMotor — LTemp | *0.01 (-0.02, 0.05)* | *0.47* | *-0.01 (-0.05, 0.02)* | *0.53* | *0.03 (-0.01, 0.07)* | *0.18* |
|  |  | RMotor — RTemp | *0.03 (-0.02, 0.09)* | *0.26* | *0.03 (-0.02, 0.07)* | *0.22* | *0.02 (-0.03, 0.08)* | *0.38* |
|  | Inter-hemispheric | LMotor — RTemp | *-0.001 (-0.03, 0.02)* | *0.91* | *-0.002 (-0.04, 0.03)* | *0.90* | *-0.02 (-0.04, 0.01)* | *0.30* |
|  |  | RMotor — LTemp | *0.005 (-0.02, 0.03)* | *0.63* | *-0.001 (-0.02, 0.02)* | *0.93* | *0.02 (-0.002, 0.04)* | *0.08* |
| Frontal to Temporal | Intra-hemispheric | LFront — LTemp | *0.006 (-0.03, 0.04)* | *0.73* | *-0.02 (-0.06, 0.02)* | *0.41* | *0.01 (-0.04, 0.07)* | *0.65* |
|  |  | RFront — RTemp | *-0.008 (-0.04, 0.03)* | *0.63* | *-0.04 (-0.08, 0.007)* | *0.10* | *-0.03 (-0.08, 0.02)* | *0.24* |
|  | Inter-hemispheric | RFront — LTemp | *0.008 (-0.03, 0.05)* | *0.70* | *-0.005 (-0.04, 0.03)* | *0.78* | *0.03 (-0.01, 0.07)* | *0.16* |
|  |  | LFront — RTemp | *-0.005 (-0.04, 0.03)* | *0.78* | *-0.01 (-0.04, 0.01)* | *0.24* | *-0.02 (-0.05, 0.003)* | *0.08* |

P-values meeting threshold (p<0.0042) are in bold. LFront: Left Inferior Frontal; LTemp: Left Superior Temporal; LMotor: Left Motor; RFront: Right Inferior Frontal; RTemp: Right Superior Temporal; RMotor: Right Motor.

**Supplementary Table 7.** Group differences in connectivity for Children with SeLECTS on and off ASM (SeLECTS+ASM vs SeLECTS-ASM) during all tasks.

|  |  | Region Pair | Verb generation | | Repetition | | Resting | |
| --- | --- | --- | --- | --- | --- | --- | --- | --- |
|  |  |  | Estimate  (95% Cl) | p-value | Estimate  (95% Cl) | p-value | Estimate  (95% Cl) | p-value |
| Motor to Frontal | Intra-hemispheric | LMotor — LFront | *0.09 (0.02, 0.16)* | *0.01* | *0.06 (0.004, 0.11)* | *0.04* | *0.08 (0.01, 0.15)* | *0.02* |
|  |  | RMotor — RFront | *0.05 (0.009, 0.10)* | *0.38* | *0.05 (0.008, 0.1)* | *0.02* | *0.05 (-0.04, 0.14)* | *0.25* |
|  | Inter-hemispheric | LMotor — RFront | *0.04 (-0.04, 0.12)* | *0.38* | *0.05 (-0.01, 0.12)* | *0.12* | *0.02 (-0.03, 0.08)* | *0.39* |
|  |  | RMotor — LFront | *0.05 (-0.005, 0.10)* | *0.07* | *0.04 (0.01, 0.08)* | *0.01* | *0.05 (-0.005, 0.11)* | *0.07* |
| Motor to Temporal | Intra-hemispheric | LMotor — LTemp | *0.05 (-0.002, 0.11)* | *0.06* | *0.04 (-0.006, 0.09)* | *0.09* | *0.02 (-0.02, 0.07)* | *0.35* |
|  |  | RMotor — RTemp | *-0.01 (-0.07, 0.05)* | *0.71* | *0.005 (-0.03, 0.04)* | *0.80* | *0.01 (-0.05, 0.07)* | *0.71* |
|  | Inter-hemispheric | LMotor — RTemp | *0.03 (-0.01, 0.08)* | *0.18* | *0.03 (-0.01, 0.08)* | *0.16* | *0.03 (-0.006, 0.06)* | *0.11* |
|  |  | RMotor — LTemp | *0.06 (0.003, 0.12)* | *0.04* | *0.03 (-0.03, 0.08)* | *0.30* | *0.03 (-0.007, 0.06)* | *0.12* |
| Frontal to Temporal | Intra-hemispheric | LFront — LTemp | *0.12 (0.04, 0.20)* | ***0.003*** | *0.1 (0.02, 0.18)* | *0.02* | *0.04 (-0.04, 0.12)* | *0.29* |
|  |  | RFront — RTemp | *0.04 (-0.02, 0.10)* | *0.17* | *0.1 (0.02, 0.17)* | *0.01* | *0.06 (-0.01, 0.13)* | *0.09* |
|  | Inter-hemispheric | RFront — LTemp | *0.07 (-0.001, 0.15)* | *0.05* | *0.05 (-0.02, 0.12)* | *0.16* | *0.004 (-0.05, 0.06)* | *0.86* |
|  |  | LFront — RTemp | *0.06 (-0.008, 0.12)* | *0.09* | *0.06 (0.02, 0.10)* | ***0.003*** | *0.06 (0.02, 0.10)* | *0.0043* |

P-values meeting threshold (p<0.0042) are in bold. LFront: Left Inferior Frontal; LTemp: Left Superior Temporal; LMotor: Left Motor; RFront: Right Inferior Frontal; RTemp: Right Superior Temporal; RMotor: Right Motor.

**Supplementary Table 8.** Group differences in connectivity for Children with SeLECTS and Controls in ALPHA band during all tasks.

|  |  | Region Pair | Verb Generation | | Repetition | | Resting | |
| --- | --- | --- | --- | --- | --- | --- | --- | --- |
|  |  |  | Estimate  (95% Cl) | p-value | Estimate  (95% Cl) | p-value | Estimate  (95% Cl) | p-value |
| Motor to Frontal | Intra-hemispheric | LMotor — LFront | *0.02 (-0.03, 0.07)* | *0.44* | *0.04 (-0.01, 0.09)* | *0.13* | *0.05 (-0.02, 0.1)* | *0.16* |
|  |  | RMotor — RFront | *0.006 (-0.04, 0.05)* | *0.78* | *0.04 (-0.02, 0.09)* | *0.20* | *0.06 (-0.009, 0.1)* | *0.09* |
|  | Inter-hemispheric | LMotor — RFront | *0.02 (-0.03, 0.07)* | *0.51* | *0.05 (-0.02, 0.1)* | *0.19* | *0.003 (-0.07, 0.08)* | *0.94* |
|  |  | RMotor — LFront | *0.03 (-0.01, 0.07)* | *0.14* | *0.03 (-0.02, 0.08)* | *0.18* | *0.06 (-0.005, 0.1)* | *0.07* |
| Motor to Temporal | Intra-hemispheric | LMotor — LTemp | *0.07 (-0.001, 0.1)* | *0.05* | *0.03 (-0.04, 0.09)* | *0.41* | *0.04 (-0.01, 0.1)* | *0.13* |
|  |  | RMotor—RTemp | *0.007 (-0.05, 0.06)* | *0.80* | *-0.001 (-0.05, 0.05)* | *0.97* | *0.05 (-0.001, 0.1)* | *0.05* |
|  | Inter-hemispheric | LMotor — RTemp | *0.02 (-0.03, 0.06)* | *0.47* | *0.03 (-0.02, 0.07)* | *0.25* | *0.006 (-0.03, 0.05)* | *0.77* |
|  |  | RMotor — LTemp | *0.07 (0.01, 0.1)* | *0.01* | *0.04 (-0.01, 0.09)* | *0.15* | *0.04 (-0.001, 0.08)* | *0.06* |
| Frontal to Temporal | Intra-hemispheric | LFront — LTemp | *0.08 (0.01, 0.1)* | *0.02* | *0.05 (-0.02, 0.1)* | *0.14* | *-0.03 (-0.09, 0.04)* | *0.38* |
|  |  | RFront — RTemp | *-0.03 (-0.1, 0.04)* | *0.42* | *-0.02 (-0.1, 0.06)* | *0.63* | *-0.01 (-0.09, 0.06)* | *0.69* |
|  | Inter-hemispheric | RFront — LTemp | *0.06 (-0.001, 0.1)* | *0.05* | *0.06 (-0.006, 0.1)* | *0.08* | *-0.01 (-0.07, 0.05)* | *0.71* |
|  |  | LFront — RTemp | *-0.02 (-0.08, 0.04)* | *0.50* | *-0.009 (-0.07, 0.05)* | *0.79* | *0.01 (-0.05, 0.08)* | *0.66* |

P-values meeting threshold (p<0.0042) are in bold. LFront: Left Inferior Frontal; LTemp: Left Superior Temporal; LMotor: Left Motor; RFront: Right Inferior Frontal; RTemp: Right Superior Temporal; RMotor: Right Motor.

**Supplementary Table 9.** Group differences in connectivity for children with SeLECTS and Controls in BETA band during all tasks.

|  |  | Region Pair | Verb Generation | | Repetition | | Resting | |
| --- | --- | --- | --- | --- | --- | --- | --- | --- |
|  |  |  | Estimate  (95% Cl) | p-value | Estimate  (95% Cl) | p-value | Estimate  (95% Cl) | p-value |
| Motor to Frontal | Intra-hemispheric | LMotor — LFront | *0.05 (-0.009, 0.1)* | *0.10* | *0.04 (-0.02, 0.09)* | *0.16* | *0.04 (-0.02, 0.09)* | *0.16* |
|  |  | RMotor — RFront | *-0.008 (-0.06, 0.04)* | *0.75* | *0.02 (-0.04, 0.09)* | *0.49* | *0.02 (-0.04, 0.09)* | *0.52* |
|  | Inter-hemispheric | LMotor — RFront | *0.06 (-0.001, 0.1)* | *0.06* | *0.06 (-0.02, 0.14)* | *0.12* | *-0.003 (-0.06, 0.06)* | *0.93* |
|  |  | RMotor — LFront | *0.05 (-0.007, 0.1)* | *0.09* | *0.06 (-0.001, 0.1)* | *0.05* | *0.03 (-0.04, 0.09)* | *0.41* |
| Motor to Temporal | Intra-hemispheric | LMotor — LTemp | *0.02 (-0.04, 0.08)* | *0.55* | *0.02 (-0.05, 0.09)* | *0.60* | *0.02 (-0.03, 0.08)* | *0.43* |
|  |  | RMotor — RTemp | *0.03 (-0.03, 0.1)* | *0.35* | *0.06 (-0.01, 0.1)* | *0.11* | *0.006 (-0.05, 0.06)* | *0.84* |
|  | Inter-hemispheric | LMotor — RTemp | *0.06 (0.009, 0.1)* | *0.02* | *0.05 (-0.01, 0.1)* | *0.11* | *0.03 (-0.02, 0.07)* | *0.22* |
|  |  | RMotor — LTemp | *0.04 (-0.01, 0.08)* | *0.15* | *0.05 (-0.02, 0.1)* | *0.14* | *0.03 (-0.01, 0.07)* | *0.18* |
| Frontal to Temporal | Intra-hemispheric | LFront — LTemp | *0.04 (-0.03, 0.1)* | *0.26* | *0.02 (-0.07, 0.1)* | *0.70* | *0.01 (-0.06, 0.09)* | *0.73* |
|  |  | RFront — RTemp | *-0.004 (-0.09, 0.08)* | *0.92* | *0.02 (-0.07, 0.1)* | *0.64* | *-0.02 (-0.1, 0.05)* | *0.54* |
|  | Inter-hemispheric | RFront — LTemp | *0.03 (-0.03, 0.09)* | *0.39* | *0.02 (-0.06, 0.1)* | *0.59* | *-0.01 (-0.08, 0.05)* | *0.70* |
|  |  | LFront — RTemp | *0.01 (-0.06, 0.08)* | *0.79* | *0.03 (-0.05, 0.1)* | *0.45* | *0.004 (-0.06, 0.06)* | *0.89* |

P-values meeting threshold (p<0.0042) are in bold. LFront: Left Inferior Frontal; LTemp: Left Superior Temporal; LMotor: Left Motor; RFront: Right Inferior Frontal; RTemp: Right Superior Temporal; RMotor: Right Motor.

**Supplementary Table 10.** Group differences in connectivity for children with SeLECTS and Controls in GAMMA band during all tasks.

|  |  | Region Pair | Verb Generation | | Repetition | | Resting | |
| --- | --- | --- | --- | --- | --- | --- | --- | --- |
|  |  |  | Estimate  (95% Cl) | p-value | Estimate  (95% Cl) | p-value | Estimate  (95% Cl) | p-value |
| Motor to Frontal | Intra-hemispheric | LMotor — LFront | *-0.01 (-0.09, 0.06)* | *0.70* | *0.01 (-0.06, 0.09)* | *0.72* | *0.03 (-0.04, 0.09)* | *0.40* |
|  |  | RMotor — RFront | *-0.01 (-0.1, 0.08)* | *0.83* | *0.01 (-0.08, 0.1)* | *0.82* | *0.03 (-0.06, 0.1)* | *0.52* |
|  | Inter-hemispheric | LMotor — RFront | *0.04 (-0.01, 0.1)* | *0.12* | *0.05 (-0.01, 0.1)* | *0.11* | *0.03 (-0.01, 0.08)* | *0.14* |
|  |  | RMotor — LFront | *0.01 (-0.04, 0.07)* | *0.66* | *0.05 (-0.02, 0.1)* | *0.13* | *0.01 (-0.05, 0.08)* | *0.69* |
| Motor to Temporal | Intra-hemispheric | LMotor — LTemp | *-0.03 (-0.1, 0.05)* | *0.49* | *-0.002 (-0.08, 0.08)* | *0.97* | *0.003 (-0.05, 0.06)* | *0.90* |
|  |  | RMotor — RTemp | *-0.03 (-0.1, 0.04)* | *0.36* | *-0.004 (-0.09, 0.08)* | *0.93* | *-0.005 (-0.08, 0.07)* | *0.90* |
|  | Inter-hemispheric | LMotor — RTemp | *0.03 (-0.03, 0.09)* | *0.31* | *0.02 (-0.04, 0.08)* | *0.48* | *0.03 (-0.02, 0.09)* | *0.18* |
|  |  | RMotor — LTemp | *0.01 (-0.05, 0.08)* | *0.72* | *0.02 (-0.05, 0.08)* | *0.62* | *0.01 (-0.03, 0.05)* | *0.64* |
| Frontal to Temporal | Intra-hemispheric | LFront — LTemp | *-0.002 (-0.09, 0.09)* | *0.96* | *-0.02 (-0.1, 0.08)* | *0.73* | *0.006 (-0.06, 0.07)* | *0.87* |
|  |  | RFront — RTemp | *-0.03 (-0.1, 0.06)* | *0.55* | *0.002 (-0.1, 0.1)* | *0.96* | *0.009 (-0.09, 0.1)* | *0.86* |
|  | Inter-hemispheric | RFront — LTemp | *0.01 (-0.04, 0.07)* | *0.71* | *0.03 (-0.05, 0.1)* | *0.43* | *0.02 (-0.06, 0.09)* | *0.63* |
|  |  | LFront — RTemp | *0.01 (-0.04, 0.07)* | *0.61* | *0.05 (-0.02, 0.1)* | *0.18* | *-0.0005 (-0.05, 0.05)* | *0.98* |

P-values meeting threshold (p<0.0042) are in bold. LFront: Left Inferior Frontal; LTemp: Left Superior Temporal; LMotor: Left Motor; RFront: Right Inferior Frontal; RTemp: Right Superior Temporal; RMotor: Right Motor.

**Supplementary Material & Figure 2. Group differences in connectivity during the verb generation task with sliding time window analysis**

**Rationale:** Our primary analyses average over a 3 second epoch, which may miss important temporally specific changes in connectivity as language processing occurs within hundreds of milliseconds. Phase-based connectivity measures require 3-5 cycles to be reliable, and hence theta connectivity requires ~1 second epochs. Here, we performed a sliding scale analysis to test whether our inferences about group or task connectivity differences changed when using more temporally precise epochs.

**Methods:** We re-segmented data from the verb generation task into shorter time windows, including the time window immediately before noun onset (-1 to 0 seconds), immediately after noun presentation (0 to 1 second), 0.5 to 1.5 seconds after noun presentation, and 1 to 2 seconds after noun presentation. We fit a GEE model with theta wPLI connectivity as the dependent variable and group (SeLECTS/controls), time (-1 to 0 second; 0 to 1 second; 0.5 to 1.5 seconds; 1 to 2 seconds), and group by time interaction as independent variables, adjusting for age & sex. We ran separate models for each of the 12 region-to-region connectivity pairs. We did not correct for multiple comparisons given this is an exploratory analysis.

**Supplementary Figure 2.** Group differences in connectivity during the verb generation task with sliding time window analysis

Estimated marginal mean connectivity with 95% confidence intervals for each group. P-values <0.05 are noted. Left column: Motor to Inferior Frontal connectivity; Middle column: Motor to Superior Temporal connectivity; Right column: Inferior Frontal to Superior Temporal connectivity.

**Conclusion:** Results were consistent with analyses using longer 3 second epochs. Inferences about group connectivity differences do not change significantly with enhanced temporal precision.

**Supplementary Table 11.** Group differences in connectivity for children with SeLECTS and Controls during all tasks adjusting for IQ and SES.

|  |  | Region Pair | Verb generation | | Repetition | | Resting | |
| --- | --- | --- | --- | --- | --- | --- | --- | --- |
|  |  |  | Estimate  (95% Cl) | p-value | Estimate  (95% Cl) | p-value | Estimate  (95% Cl) | p-value |
| Motor to Frontal | Intra-hemispheric | LMotor — LFront | *-0.07*  *(-0.14, -0.01)* | *0.03* | *-0.06*  *(-0.11, -0.01)* | *0.02* | *-0.06*  *(-0.13, 0.00)* | *0.06* |
|  |  | RMotor — RFront | *-0.05*  *(-0.09, -0.01)* | *0.01* | *-0.05*  *(-0.08, -0.01)* | *0.01* | *-0.07*  *(-0.13, -0.01)* | *0.01* |
|  | Inter-hemispheric | LMotor — RFront | *-0.05*  *(-0.09, -0.01)* | *0.02* | *-0.05*  *(-0.09, -0.01)* | *0.01* | *-0.02*  *(-0.06, 0.02)* | *0.37* |
|  |  | RMotor — LFront | *-0.05*  *(-0.09, 0.00)* | *0.06* | *-0.04*  *(-0.07, -0.01)* | *0.01* | *-0.05*  *(-0.09, -0.01)* | *0.01* |
| Motor to Temporal | Intra-hemispheric | LMotor — LTemp | ***-0.07***  ***(-0.11, -0.03)*** | ***0.002*** | *-0.03*  *(-0.07, 0.01)* | *0.14* | *-0.05*  *(-0.09, -0.01)* | *0.01* |
|  |  | RMotor — RTemp | *-0.01*  *(-0.06, 0.04)* | *0.67* | *-0.02*  *(-0.06, 0.02)* | *0.25* | *-0.02*  *(-0.06, 0.03)* | *0.50* |
|  | Inter-hemispheric | LMotor — RTemp | *-0.01*  *(-0.05, 0.02)* | *0.45* | *-0.02*  *(-0.06, 0.02)* | *0.26* | *0.00*  *(-0.03, 0.04)* | *0.75* |
|  |  | RMotor — LTemp | *-0.04*  *(-0.08, -0.01)* | *0.02* | *-0.03*  *(-0.07, 0.01)* | *0.17* | *-0.03*  *(-0.05, -0.01)* | *0.01* |
| Frontal to Temporal | Intra-hemispheric | LFront — LTemp | *-0.09*  *(-0.16, -0.02)* | *0.01* | *-0.07*  *(-0.15, -0.00)* | *0.04* | *-0.05*  *(-0.11, 0.02)* | *0.17* |
|  |  | RFront — RTemp | *0.01*  *(-0.04, 0.05)* | *0.75* | *-0.00*  *(-0.07, 0.06)* | *0.90* | *0.01*  *(-0.04, 0.07)* | *0.71* |
|  | Inter-hemispheric | RFront — LTemp | *-0.05*  *(-0.11, -0.00)* | *0.04* | *-0.03*  *(-0.08, 0.03)* | *0.33* | *-0.03*  *(-0.07, -0.00)* | *0.05* |
|  |  | LFront — RTemp | *-0.04*  *(-0.09, 0.02)* | *0.18* | *-0.03*  *(-0.06, 0.01)* | *0.15* | *-0.01*  *(-0.05, 0.02)* | *0.46* |

P-values meeting threshold (p<0.0042) are in bold. LFront: Left Inferior Frontal; LTemp: Left Superior Temporal; LMotor: Left Motor; RFront: Right Inferior Frontal; RTemp: Right Superior Temporal; RMotor: Right Motor.

**Supplementary Table 12.** Association between clinical language performance and connectivity in children with SeLECTS adjusting for age, sex, socioeconomic status, IQ, and handedness.

|  |  | Region Pair | Verb generation | | Repetition | | Resting | |
| --- | --- | --- | --- | --- | --- | --- | --- | --- |
|  |  |  | Estimate  (95% Cl) | p-value | Estimate  (95% Cl) | p-value | Estimate  (95% Cl) | p-value |
| Motor to Frontal | Intra-hemispheric | LMotor — LFront | *-23.5 (-60.0, 12.9)* | *0.21* | *-22.4 (-65.2, 20.4)* | *0.3* | *-41.9 (-80.0, -3.8)* | *0.03* |
|  |  | RMotor — RFront | *-72.8 (-137.8, -7.7)* | *0.03* | *-54.1 (-138.9, 30.7)* | *0.21* | *-21.0 (-49.1, 7.0)* | *0.14* |
|  | Inter-hemispheric | LMotor — RFront | *-55.3 (-103.2, -7.3)* | *0.02* | *-49.5 (-107.8, 8.9)* | *0.1* | *14.2 (-43.4, 71.8)* | *0.63* |
|  |  | RMotor — LFront | *-39.2 (-98.2, 19.8)* | *0.19* | *-61.2 (-173.1, 50.8)* | *0.28* | *-17.9 (-54.7, 19.0)* | *0.34* |
| Motor to Temporal | Intra-hemispheric | LMotor — LTemp | *0.74 (-64.2, 65.7)* | *0.98* | *2.7 (-80.7, 86.2)* | *0.95* | *33.9 (-33.2, 100.9)* | *0.32* |
|  |  | RMotor — RTemp | *-15.8 (-92.2, 60.7)* | *0.69* | *-39.1 (-142.8, 64.6)* | *0.46* | *28.8 (-21.1, 78.7)* | *0.26* |
|  | Inter-hemispheric | LMotor — RTemp | *-64.2 (-101.6, -26.9)* | ***0.0008*** | *-47.5 (-105.9, 11.0)* | *0.11* | *1.0 (-61.7, 63.8)* | *0.97* |
|  |  | RMotor — LTemp | *-62.0 (-84.4, -39.6)* | ***0.0001*** | *-52.7 (-77.6, -27.8)* | ***0.0001*** | *77.7 (-22.1, 177.4)* | *0.13* |
| Frontal to Temporal | Intra-hemispheric | LFront — LTemp | *-29.4 (-59.8, 1.0)* | *0.06* | *-27.5 (-54.8, -0.29)* | *0.05* | *-9.9 (-39.6, 19.8)* | *0.51* |
|  |  | RFront — RTemp | *-62.2 (-110.0, -14.5)* | *0.01* | *-44.2 (-62.7, -25.6)* | ***0.0001*** | *-11.6 (-34.6, 11.4)* | *0.32* |
|  | Inter-hemispheric | RFront — LTemp | *-47.1 (-70.2, -24.0)* | ***0.0001*** | *-39.6 (-58.7, -20.5)* | ***0.0001*** | *45.2 (-7.7, 98.1)* | *0.09* |
|  |  | LFront — RTemp | *-48.5 (-72.9, -24.1)* | ***0.0001*** | *-62.8 (-128.4, 2.9)* | *0.06* | *-45.9 (-111.2, 19.3)* | *0.17* |

P-values meeting threshold (p<0.0042) are in bold. LFront: Left Inferior Frontal; LTemp: Left Superior Temporal; LMotor: Left Motor; RFront: Right Inferior Frontal; RTemp: Right Superior Temporal; RMotor: Right Motor.

**Supplementary Table 13.** Association between clinical language performance and connectivity in Controls adjusting for age, sex, socioeconomic status, IQ, and handedness.

|  |  | Region Pair | Verb generation | | Repetition | | Resting | |
| --- | --- | --- | --- | --- | --- | --- | --- | --- |
|  |  |  | Estimate  (95% Cl) | p-value | Estimate  (95% Cl) | p-value | Estimate  (95% Cl) | p-value |
| Motor to Frontal | Intra-hemispheric | LMotor — LFront | *-58.8 (-105.7, -11.9)* | *0.01* | *-38.6 (-100.1, 22.9)* | *0.22* | *-46.6 (-93.7, 0.5)* | *0.05* |
|  |  | RMotor — RFront | *-70.1 (-157.9, 17.7)* | *0.12* | *-87.3 (-216.4, 41.8)* | *0.19* | *-84.6 (-152.4, -16.8)* | *0.01* |
|  | Inter-hemispheric | LMotor — RFront | *-39.0 (-132.0, 54.1)* | *0.41* | *6.5 (-78.9, 91.9)* | *0.88* | *-31.5 (-84.7, 21.7)* | *0.25* |
|  |  | RMotor — LFront | *-24.6 (-105.8, 56.6)* | *0.55* | *-88.2 (-199.9, 23.4)* | *0.12* | *-81.8 (-149.5, -14.1)* | *0.02* |
| Motor to Temporal | Intra-hemispheric | LMotor — LTemp | *-62.4 (-165.1, 40.4)* | *0.23* | *-28.9 (-87.2, 29.3)* | *0.33* | *37.4 (-15.5, 90.3)* | *0.17* |
|  |  | RMotor — RTemp | *-28.1 (-70.7, 14.4)* | *0.19* | *-24.3 (-75.9, 27.3)* | *0.36* | *-55.6 (-82.2, -29.0)* | ***0.0001*** |
|  | Inter-hemispheric | LMotor — RTemp | *-13.4 (-142.1, 115.2)* | *0.84* | *-64.0 (-104.6, -23.4)* | ***0.002*** | *-67.8 (-106.5, -29.0)* | ***0.0006*** |
|  |  | RMotor — LTemp | *-104.8 (-175.0, -34.6)* | ***0.003*** | *-123.0 (-215.5, -30.4)* | *0.009* | *-84.4 (-278.1, 109.2)* | *0.39* |
| Frontal to Temporal | Intra-hemispheric | LFront — LTemp | *-2.0 (-84.7, 80.7)* | *0.96* | *-1.1 (-46.2, 43.9)* | *0.96* | *-3.5 (-51.7, 44.7)* | *0.89* |
|  |  | RFront — RTemp | *-32.5 (-91.4, 26.4)* | *0.28* | *-34.8 (-78.8, 9.2)* | *0.12* | *-45.8 (-77.7, -14.0)* | *0.005* |
|  | Inter-hemispheric | RFront — LTemp | *-14.3 (-123.4, 94.8)* | *0.80* | *12.1 (-71.4, 95.5)* | *0.78* | *-1.2 (-82.2, 79.8)* | *0.98* |
|  |  | LFront — RTemp | *-57.1 (-124.5, 10.3)* | *0.10* | *-75.5 (-142.9, -8.1)* | *0.03* | *-79.8 (-127.5, -32.1)* | ***0.001*** |

P-values meeting threshold (p<0.0042) are in bold. LFront: Left Inferior Frontal; LTemp: Left Superior Temporal; LMotor: Left Motor; RFront: Right Inferior Frontal; RTemp: Right Superior Temporal; RMotor: Right Motor.

**Supplementary Figure 3.** Association between clinical language performance and connectivity in children with SeLECTS and Controls during the verb generation task with marked influential case.

Scatter plots show mean connectivity vs. CTOPP-2 Composite scores with estimated marginal fit lines and 95% confidence intervals. P-values meeting threshold (p<0.0042) noted and color coded to group (red=SeLECTS; blue=controls). Left column: Motor to Inferior Frontal connectivity; Middle column: Motor to Superior Temporal connectivity; Right column: Inferior Frontal to Superior Temporal connectivity. Sensitivity analyses excluding one influential case with extreme connectivity values attenuated these associations (circled in black; Supplementary Table 9).

**Supplementary Figure 4.** Association between clinical language performance and connectivity in children with SeLECTS and Controls during the repetition task with marked influential case.

Scatter plots of mean connectivity vs. CTOPP-2 Composite scores for each subject and estimated marginal fit lines with 95% confidence intervals. P-values meeting threshold (p<0.0042) noted and color coded to group (red=SeLECTS; blue=controls). Left column: Motor to Inferior Frontal; Middle column: Motor to Superior Temporal; Right column: Inferior Frontal to Superior Temporal. Sensitivity analyses excluding one influential case with extreme connectivity values attenuated these associations (circled in black; Supplementary Table 9).

**Supplementary Figure 5.** Association between clinical language performance and connectivity in children with SeLECTS and Controls during the resting task with marked influential case.

Scatter plots of mean connectivity vs. CTOPP-2 Composite scores for each subject and estimated marginal fit lines with 95% confidence intervals. P-values meeting threshold (p<0.0042) noted and color coded to group (red=SeLECTS; blue=controls). Left column: Motor to Inferior Frontal; Middle column: Motor to Superior Temporal; Right column: Inferior Frontal to Superior Temporal. Sensitivity analyses excluding one influential case with extreme connectivity values attenuated these associations (circled in black; Supplementary Table 9).

**Supplementary Table 14.** Group differences in connectivity for children with SeLECTS and Controls during all tasks without potential outlier.

|  |  | Region Pair | Verb Generation | | Repetition | | Resting | |
| --- | --- | --- | --- | --- | --- | --- | --- | --- |
|  |  |  | Estimate  (95% Cl) | p-value | Estimate (95% Cl) | p-value | Estimate (95% Cl) | p-value |
| Motor to Frontal | Intra-  hemispheric | LMotor — LFront | *0.04*  *(-0.01, 0.09)* | *0.12* | *0.03*  *(-0.009, 0.07)* | *0.14* | *0.04*  *(-0.01, 0.09)* | *0.14* |
|  |  | RMotor — RFront | *0.05*  *(0.03, 0.08)* | ***0.0002*** | *0.06*  *(0.03, 0.08)* | ***<0.0001*** | *0.07*  *(0.02, 0.12)* | *0.01* |
|  | Inter-  hemispheric | LMotor — RFront | *0.06*  *(0.01, 0.10)* | *0.008* | *0.06*  *(0.02, 0.09)* | ***0.0008*** | *0.02*  *(-0.01, 0.06)* | *0.18* |
|  |  | RMotor — LFront | *0.03*  *(-0.006, 0.06)* | *0.11* | *0.03*  *(0.01, 0.06)* | *0.005* | *0.04*  *(0.006, 0.08)* | *0.02* |
| Motor to Temporal | Intra-  hemispheric | LMotor — LTemp | *0.04*  *(0.0003, 0.07)* | *0.05* | *0.006*  *(-0.02, 0.04)* | *0.67* | *0.04*  *(0.01, 0.07)* | *0.009* |
|  |  | RMotor — RTemp | *0.02*  *(-0.02, 0.06)* | *0.39* | *0.03*  *(-0.009, 0.06)* | *0.14* | *0.03*  *(-0.007, 0.07)* | *0.11* |
|  | Inter-  hemispheric | LMotor — RTemp | *0.008*  *(-0.01, 0.03)* | *0.50* | *0.009*  *(-0.02, 0.04)* | *0.53* | *0.0008*  *(-0.03, 0.03)* | *0.95* |
|  |  | RMotor — LTemp | *0.03*  *(-0.006, 0.06)* | *0.11* | *0.004*  *(-0.01, 0.02)* | *0.66* | *0.03*  *(0.01, 0.06)* | ***0.0008*** |
| Frontal to Temporal | Intra-  hemispheric | LFront — LTemp | *0.06*  *(0.02, 0.11)* | *0.008* | *0.02*  *(-0.02, 0.07)* | *0.31* | *0.04*  *(-0.009, 0.09)* | *0.11* |
|  |  | RFront — RTemp | *0.008*  *(-0.02, 0.04)* | *0.60* | *0.005*  *(-0.03, 0.04)* | *0.80* | *0.007*  *(-0.04, 0.06)* | *0.78* |
|  | Inter-  hemispheric | RFront — LTemp | *0.04*  *(0.003, 0.07)* | *0.03* | *0.01*  *(-0.02, 0.04)* | *0.44* | *0.04*  *(0.005, 0.07)* | *0.02* |
|  |  | LFront — RTemp | *0.02*  *(-0.01, 0.04)* | *0.29* | *0.01*  *(-0.01, 0.04)* | *0.30* | *0.01*  *(-0.02, 0.04)* | *0.56* |

P-values meeting threshold (p<0.0042) are in bold. LFront: Left Inferior Frontal; LTemp: Left Superior Temporal; LMotor: Left Motor; RFront: Right Inferior Frontal; RTemp: Right Superior Temporal; RMotor: Right Motor.

**Supplementary Table 15.** Association between clinical language performance and connectivity in children with SeLECTS without potential outlier.

|  |  | Region Pair | Verb Generation | | Repetition | | Resting | |
| --- | --- | --- | --- | --- | --- | --- | --- | --- |
|  |  |  | Estimate  (95% Cl) | p-value | Estimate  (95% Cl) | p-value | Estimate  (95% Cl) | p-value |
| Motor to Frontal | Intra-  hemispheric | LMotor — LFront | *-7.2*  *(-39.5, 25.1)* | *0.66* | *-16.4*  *(-55.7, 22.9)* | *0.41* | *-41.8*  *(-79.3, -4.4)* | *0.03* |
|  |  | RMotor — RFront | *-19.4*  *(-86.6, 47.8)* | *0.57* | *-63.8*  *(-116.6, -11.1)* | *0.02* | *-10.8*  *(-35.6, 13.9)* | *0.39* |
|  | Inter-  hemispheric | LMotor — RFront | *-54.6*  *(-110.3, 1.0)* | *0.05* | *-44.1*  *(-84.7, -3.5)* | *0.03* | *-12.0*  *(-73.2, 49.2)* | *0.70* |
|  |  | RMotor — LFront | *-23.5*  *(-88.7, 41.7)* | *0.48* | *-91.1*  *(-188.1, 5.9)* | *0.07* | *-32.9*  *(-67.1, 1.4)* | *0.06* |
| Motor to Temporal | Intra-  hemispheric | LMotor — LTemp | *0.02*  *(-56.1, 56.2)* | *0.99* | *51.8*  *(-18.4, 122.0)* | *0.15* | *-2.9*  *(-65.6, 59.8)* | *0.93* |
|  |  | RMotor—RTemp | *31.4*  *(-29.8, 92.6)* | *0.32* | *5.7*  *(-79.3, 90.8)* | *0.89* | *1.9*  *(-56.2, 60.1)* | *0.95* |
|  | Inter-  hemispheric | LMotor — RTemp | *-39.1*  *(-124.8, 46.6)* | *0.37* | *-10.8*  *(-88.3, 66.7)* | *0.78* | *-64.6*  *(-131.7, 2.6)* | *0.06* |
|  |  | RMotor — LTemp | *-49.6*  *(-121.9, 22.6)* | *0.18* | *0.88*  *(-141.0, 142.8)* | *0.99* | *-45.5*  *(-118.9, 27.8)* | *0.22* |
| Frontal to Temporal | Intra-  hemispheric | LFront — LTemp | *-22.5*  *(-54.6, 9.6)* | *0.17* | *-16.7*  *(-44.0, 10.6)* | *0.23* | *-27.4*  *(-54.3, -0.47)* | *0.05* |
|  |  | RFront — RTemp | *-10.9*  *(-49.9, 28.0)* | *0.58* | *-36.7*  *(-78.0, 4.5)* | *0.08* | *-23.4*  *(-40.0, -6.9)* | *0.006* |
|  | Inter-  hemispheric | RFront — LTemp | *-61.7*  *(-111.3, -12.1)* | *0.01* | *-42.0*  *(-127.5, 43.5)* | *0.34* | *0.91*  *(-62.2, 64.0)* | *0.98* |
|  |  | LFront — RTemp | *-23.4*  *(-81.1, 34.3)* | *0.43* | *-26.6*  *(-120.3, 67.2)* | *0.58* | *-69.8*  *(-124.9, -14.8)* | *0.01* |

P-values meeting threshold (p<0.0042) are in bold. LFront: Left Inferior Frontal; LTemp: Left Superior Temporal; LMotor: Left Motor; RFront: Right Inferior Frontal; RTemp: Right Superior Temporal; RMotor: Right Motor.
